# The ConDialInt Model: Condensation, Dialogality, and Intentionality Dimensions of Inner Speech Within a Hierarchical Predictive Control Framework

**DOI:** 10.3389/fpsyg.2019.02019

**Published:** 2019-09-18

**Authors:** Romain Grandchamp, Lucile Rapin, Marcela Perrone-Bertolotti, Cédric Pichat, Célise Haldin, Emilie Cousin, Jean-Philippe Lachaux, Marion Dohen, Pascal Perrier, Maëva Garnier, Monica Baciu, Hélène Lœvenbruck

**Affiliations:** ^1^Univ. Grenoble Alpes, Univ. Savoie Mont Blanc, CNRS, LPNC, Grenoble, France; ^2^INSERM U1028, CNRS UMR5292, Brain Dynamics and Cognition Team, Lyon Neurosciences Research Center, Bron, France; ^3^Univ. Grenoble Alpes, CNRS, Grenoble INP, GIPSA-lab, Grenoble, France

**Keywords:** inner speech, auditory verbal imagery, mind wandering, condensation, dialogality, intentionality, fMRI, predictive control

## Abstract

Inner speech has been shown to vary in form along several dimensions. Along condensation, condensed inner speech forms have been described, that are supposed to be deprived of acoustic, phonological and even syntactic qualities. Expanded forms, on the other extreme, display articulatory and auditory properties. Along dialogality, inner speech can be monologal, when we engage in internal soliloquy, or dialogal, when we recall past conversations or imagine future dialogs involving our own voice as well as that of others addressing us. Along intentionality, it can be intentional (when we deliberately rehearse material in short-term memory) or it can arise unintentionally (during mind wandering). We introduce the ConDialInt model, a neurocognitive predictive control model of inner speech that accounts for its varieties along these three dimensions. ConDialInt spells out the condensation dimension by including inhibitory control at the conceptualization, formulation or articulatory planning stage. It accounts for dialogality, by assuming internal model adaptations and by speculating on neural processes underlying perspective switching. It explains the differences between intentional and spontaneous varieties in terms of monitoring. We present an fMRI study in which we probed varieties of inner speech along dialogality and intentionality, to examine the validity of the neuroanatomical correlates posited in ConDialInt. Condensation was also informally tackled. Our data support the hypothesis that expanded inner speech recruits speech production processes down to articulatory planning, resulting in a predicted signal, the inner voice, with auditory qualities. Along dialogality, covertly using an avatar’s voice resulted in the activation of right hemisphere homologs of the regions involved in internal own-voice soliloquy and in reduced cerebellar activation, consistent with internal model adaptation. Switching from first-person to third-person perspective resulted in activations in precuneus and parietal lobules. Along intentionality, compared with intentional inner speech, mind wandering with inner speech episodes was associated with greater bilateral inferior frontal activation and decreased activation in left temporal regions. This is consistent with the reported subjective evanescence and presumably reflects condensation processes. Our results provide neuroanatomical evidence compatible with predictive control and in favor of the assumptions made in the ConDialInt model.

## Introduction

### Three Dimensions of Inner Speech

Inner language can be defined as the subjective experience of verbalization in the absence of overt articulation or sign (Alderson-Day and Fernyhough, 2015). It can be produced independently of overt speech. It contributes to enriching and shaping our inner existence and is instrumental in the maintenance of a coherent self-narrative (Perrone-Bertolotti et al., 2014; Lœvenbruck, 2018). Given the scarcity of data on inner sign language production (but see e.g., Max, 1937; McGuire et al., 1997; MacSweeney et al., 2008 and references in Lœvenbruck et al., 2018) the present article is restricted to the description of inner speech, although most of the theoretical principles we endorse presumably also apply to inner sign.

The cognitive functions (or rather uses) of inner speech have been investigated by means of introspective questionnaires and behavioral methods, in typical and atypical populations (for reviews, see e.g., Perrone-Bertolotti et al., 2014; Alderson-Day and Fernyhough, 2015; Martínez-Manrique and Vicente, 2015; Alderson-Day et al., 2018; and the volume edited by Langland-Hassan and Vicente, 2018). Previous works suggest that inner speech plays an important role in many cognitive operations, including working memory (Baddeley, 1992; Marvel and Desmond, 2012), autobiographical and prospective memory (Meacham, 1979; Conway, 2005; Morin and Hamper, 2012; Pavlenko, 2014), orientation and spatial reasoning (Loewenstein and Gentner, 2005), mental arithmetics (Sokolov, 1972), executive control (Emerson and Miyake, 2003; Laurent et al., 2016), complex problem solving (Sokolov, 1972; Baldo et al., 2005, 2015), and theory of mind judgment (Newton and de Villiers, 2007). It has also been considered that inner speech serves metacognitive functions. By making our thoughts auditorily salient (in expanded varieties of covert speech, see below), inner speaking makes us aware of our thinking processes and allows us to focus our attention on our thoughts and activities. This metacognitive ability in turn contributes to our taking perspectives on self and others and to generate self-knowledge. It has thus been suggested that inner speech fosters metacognition (Vygotsky, 1934/1986; Carruthers, 2002; Clark, 2002; Martínez-Manrique and Vicente, 2010; Jackendoff, 2011; Langland-Hassan et al., 2017), self-regulation and self-motivation (Hardy, 2006; Clowes, 2007), and self-awareness (Peirce, 1934; Vygotsky, 1934/1986; Ricœur, 1990; Dennett, 1991; Merleau-Ponty, 1948/2002; Wiley, 2006b; Morin et al., 2011; Wilkinson and Fernyhough, 2017). This diversity of uses comes with a plurality of forms. It has been suggested that inner speech varies along several dimensions (McCarthy-Jones and Fernyhough, 2011). This article seeks to provide an integrative description of these dimensions, which accounts for the occurrence of various inner speech forms.

A first dimension along which inner speech can vary is condensation. Overt speech production is classically viewed as involving three main stages: conceptualization, formulation and articulation (e.g., Dell, 1986, 2013; Bock, 1987; Kempen and Hoenkamp, 1987; Levelt, 1989). Conceptual preparation consists in planning an utterance’s meaning and purpose. The preverbal message that results can be described as highly condensed in form. Formulation translates the condensed preverbal message delivered by the conceptualizer into a linguistic structure. Formulation includes prosodic, syntactic and morpho-phonological encoding. It ends up in the sketching of a phonetic goal (or plan), expressed in a less condensed (semi-expanded) form. The articulation stage follows, consisting of articulatory planning, then execution, with full elaboration and expansion. Covert speech has been conceived of as truncated overt speech, but the stage at which the production process is interrupted is still debated. According to some scholars, inner language predominantly pertains to semantics and is unconcerned with phonological, phonetic, articulatory or auditory representations (see e.g., Vygotsky, 1934/1986; MacKay, 1992; Oppenheim and Dell, 2008, 2010). Vygotsky, for instance, claims that syntax in inner speech is maximally simplified and can be elliptical, with the omission of words and an extreme condensation of meaning. In his view, inner speech, is highly predicated, in the sense that only the necessary information is supplied. In line with Vygotsky’s view that inner speech precedes word-level formulation, Knobloch (1984, p. 230, cited by Friedrich, 2001), posits that inner language is the preliminary form of all overt language utterances. It is the mechanism by which quasi-linguistic material are supplied to semantico-syntactic processes, in a “condensed, compact and indicative form.” In this view, inner speech can therefore be conceived of as the conceptual message, cast in a pre-linguistic compact form, before formulation and articulatory planning take place. Bergounioux (2001, p. 120) likewise states that inner speech generally employs asyndeton (the omission of coordinating conjunctions), anaphora (the use of expressions whose interpretations depend on the context) and predication (the use of expressions in which only the predicate, not the subject, is formulated). In the same vein, Wiley (2006a) argues that the “syntax of inner speech is abbreviated and simplified” (p. 321) and that its semantics is also condensed, with fewer words used relative to overt language, given that key words may be used, that carry “large numbers of words or their possible meanings” (p. 323). These introspective observations of condensation are supported by several psycholinguistic experiments on the relative rates of overt and covert speech (e.g., Korba, 1990; but see Netsell et al., 2016) or on the different biases exhibited by speech slips in overt and covert modes (Oppenheim and Dell, 2008, 2010; but see Corley et al., 2011). These empirical findings suggest that, compared with overt public speech, inner language is sketchy and can be viewed as abbreviated or condensed, at the syntactic, lexical, and even phonological levels. Such condensation implies that the formulation and articulation stages may be suppressed or limited in inner language.

An alternative view is that inner speech is a simulation of overt speech production, encompassing all its stages, only interrupted prior to motor execution. In this view, inner speech entails phonological and articulatory specification and is associated with the subjective experience of a voice percept (see e.g., Postma and Noordanus, 1996; Corley et al., 2011; Scott et al., 2013). Several empirical arguments for the proposition that inner speech involves multisensory representations, together with the recruitment of the speech motor system, are provided in Lœvenbruck et al. (2018). These include psycholinguistic data, such as the verbal transformation effect (Reisberg et al., 1989; Smith et al., 1995; Sato et al., 2004) as well as electromyographical findings (McGuigan and Dollins, 1989; Nalborczyk et al., 2017) and neuroimaging data (Lœvenbruck et al., 2005; Perrone-Bertolotti et al., 2012; Yao et al., 2012; Vercueil and Perrone-Bertolotti, 2013; Kell et al., 2017). These data, in turn, suggest that inner speech may well possess many of the properties of overt speech, including its articulatory specification.

These two views can be reconciled if various degrees of unfolding of inner speech are considered. Building on the Vygotskian’s view of inner speech as the outcome of a developmental process, Fernyhough (2004, see also Geva et al., 2011; Alderson-Day and Fernyhough, 2015) has suggested that inner speech varies between two extremes. The first one, which he calls “expanded inner speech,” is claimed to correspond to an early developmental stage of inner speech, which (according to Vygotsky, 1934/1986) is an internalization of overt dialog and which includes turn-taking qualities as well as syntactic, lexical and phonological properties. The other extreme, referred to as “condensed inner speech,” is argued to correspond to Vygotsky (1934/1986) description of the latest developmental form of inner speech, which has lost most of the acoustic and structural qualities of overt speech. Fernyhough (2004) has suggested that inner speech varies with cognitive demands and emotional conditions between these two extreme forms. A similar position is taken by Vicente and Martínez-Manrique (2016), who conceive of unsymbolized thinking (as described by Hurlburt et al., 2013) as the most condensed form of inner speech and as in continuity with expanded forms of inner speech. Therefore, the two views of inner speech (abbreviation vs. simulation) can be construed as descriptions of two opposite poles on the condensation dimension. The fully condensed form only involves the highest linguistic level (semantics), and has lost most of the acoustic, phonological and even syntactic qualities of overt speech. Expanded inner speech, on the other hand, presumably engages all linguistic levels down to articulatory planning and the perception of an inner voice. It retains many of the phonological and phonetic properties of overt speech. Between the fully condensed form (preverbal message) and the expanded articulation-ready form, it can be assumed that various semi-condensed forms may exist, depending on the level at which the speech production process is truncated.

A second dimension is dialogality. As argued by Fernyhough (2004) or Jones and Fernyhough (2007a), inner speech may be considered as “irreducibly dialogic,” in that it results from a gradual process of internalization of dialogs, in which differing perspectives on the world are held and self-regulated (but see Gregory, 2017 for a slightly different view). In the Vygotskian developmental approach taken by Fernyhough, a child’s first utterances are set within external dialogs with their caregivers. Later in development, the utterances remain dialogic, with the child overtly producing both questions and answers, in an egocentric fashion (private speech, speech directed toward the self). In the last developmental stage, these dialogs become fully internalized into inner speech. Yet, even though self-directed speech may become fully internalized, Fernyhough (2004) claims that it retains the dialogic character of overt dialog, with the ability to hold differing attitudes or views on reality. In French pragmatics, a distinction is made between *dialogal discourse* in which two distinct speakers are involved, in an interpersonal way, and *dialogic discourse*, where two points of view are confronted (for the distinction between dialogic and dialogal, see Roulet, 1984; Bres, 2005; Roulet and Green, 2006). *Dialogal discourse* occurs in a communicative interaction whereas *dialogic discourse* occurs in a reflexive argumentation. An overt discourse can be “monologal dialogic,” when it is uttered by one speaker who, asserts, refutes, questions. In other words, it can be an argumented soliloquy. A discourse can also be “dialogal monologic,” when two speakers convey a single view, with no alternative. It can then be described as a unitary conversation (Maingueneau, 2016). Although it may be considered that inner speech is dialogic *in content*, since multiple perspectives can be entertained internally, we claim that it can be either monologal (soliloquial) or dialogal *in form*. Monologal inner speech occurs when we engage in internal soliloquy. In monologal situations, we can use our own voice or we can also covertly imitate someone speaking, which means we can produce internal soliloquy in another person’s voice, yet we primarily are the speaker (although obviously also the listener), and only one voice is controlled and monitored. Dialogal inner speech occurs when we imagine hearing someone, what is often referred to as auditory verbal imagery (Shergill et al., 2001). In dialogal situations, when we imagine someone talking to us, with their own voice, we primarily are the addressee (although perhaps also the speaker). This happens for instance when we recollect past dialogs or when we practice future conversations. Dialogal inner speech involves the representation and monitoring of our own voice as well as those of other people. It also sometimes requires the ability to entertain differing perspectives (Fernyhough, 2004; Jones and Fernyhough, 2007a). Therefore, we claim that inner speech can vary between two extremes: internal monolog or soliloquy – i.e., inner speaking using own voice (“Self”) – and internal dialog, which includes inner speaking and imagining others speaking with their voices (“Self and Other”). Imitative soliloquy, or monolog with another voice as one’s own, can be conceived of as lying between these two extremes. Our model seeks to account for these three distinct situations: inner speaking as self, inner speaking as modified self, inner speaking as self and other.

A third dimension is intentionality. We sometimes deliberately engage in inner speech (when we rehearse material in short-term memory), what can be called willful or intentional inner speech. Other times, we find ourselves unintentionally using inner language, what has been called verbal mind wandering (Perrone-Bertolotti et al., 2014) or spontaneously occurring inner speech (Hurlburt et al., 2016). Verbal mind wandering has been described as evanescent, fading (Egger, 1881; Saint-Paul, 1892; Hurlburt, 2011; Smadja, 2018) and its auditory qualities are often reported as fainter than that of intentional inner speech (Lœvenbruck et al., 2018).

**FIGURE 1:**
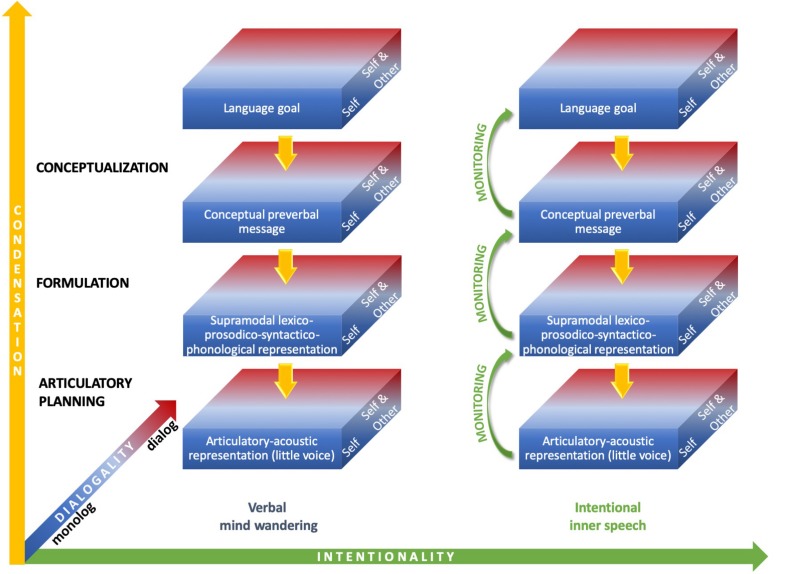
Varieties of inner speech along condensation, dialogality, and intentionality dimensions. On the vertical condensation axis, the most condensed forms **(top box)** only engage the highest linguistic level (semantics), whereas the most expanded forms engage all linguistic levels down to articulatory planning and the perception of an inner voice **(bottom box)**. On the dialogality dimension, inner speech can vary between two extremes: internal monolog or soliloquy with own voice (“Self”) and internal dialog, which includes inner speaking and imagining others speaking with their voices (“Self and Other”). Monolog with another voice as one’s own lies in between these two extremes. On the horizontal intentionality axis, inner speech can vary between verbal mind wandering, on the **left**, and intentional inner speech, on the **right**.

As depicted in Figure 1, inner speech can therefore vary along condensation, dialogality and intentionality dimensions. It can be assumed that the expanded forms most frequently arise during intentional inner speech (verbal mind wandering is often reported as fading and fleeting), but this is debatable, as unintentional varieties with expanded, audible, forms have been reported (Hurlburt, 2011).

### Monitoring of Multidimensional Inner Speech Varieties

The question of monitoring during inner speech is still an open one. Overt language production relies on verbal self-monitoring, a mechanism which allows us to control and regulate our own language productions. We can detect errors or disruptions from our initial language goals, and even correct for these errors online, sometimes even before articulation takes place (Levelt, 1983; Postma, 2000; Huettig and Hartsuiker, 2010). In many psycholinguistic models of overt speech production (e.g., Laver, 1980; Levelt, 1989), errors are detected by monitoring and parsing the phonetic plan, also called “inner speech,” prior to articulation. In our view, as described above, inner speech production is embedded in overt speech production. It engages speech production mechanisms, which can be interrupted at different stages, according to the degree of condensation. The mechanisms by which errors can be anticipated online during overt speech production are therefore engaged during inner speech production. This implies that errors in inner speech can be detected using these mechanisms. Introspective accounts suggest indeed that inner speech itself can be monitored (Bergounioux, 2001). Evidence for inner speech monitoring can be found in psycholinguistic data. Studies of inner recitation of tongue-twisters show that speech errors can be detected, even in a covert mode (e.g., Dell and Repka, 1992; Nooteboom, 2005; Oppenheim and Dell, 2008, 2010; Corley et al., 2011). The Verbal Transformation Effect (VTE) refers to the perceptual phenomenon in which listeners report hearing a new speech percept when an ambiguous stimulus is repeated rapidly (Warren, 1961). It has been shown to also occur in a covert mode (Reisberg et al., 1989; Smith et al., 1995; Sato et al., 2004). These studies suggest that inner speech alterations can be monitored, at least when participants are asked to do so. The level at which inner slips are detected is debated, however. Tongue-twister inner recitation studies suggest that errors are detected at the phonological (formulation) level. Oppenheim and Dell (2008; 2010), for instance, observed a lexical bias, which reveals that phonological representations are monitored. They found that the errors reported by the participants, when covertly repeating tongue-twisters, tend to produce more words than non-words (“reef” replaced by “leaf” is more likely than “wreath” replaced by “leath”). In overt speech, in addition to the lexical bias, a phonemic similarity effect is observed, i.e., a tendency for slips to involve similarly articulated phonemes (“reef” slips more often to “leaf,” with /r/ and /l/ sharing voicing and approximant features, than “beef,” with /r/ and /b/ only sharing voicing). This effect relies on subphonemic, articulatory representations. The covert speech errors reported by the participants in Oppenheim and Dell’s experiments do not exhibit this effect. These findings therefore suggest that monitoring for errors occurs at the formulation stage, not at the articulatory planning stage. Corley et al. (2011), however, did observe a phonemic similarity effect in the errors reported by the participants in their own tongue-twister recitation experiment. This suggests that inner slips could in fact be detected at the articulation planning level. In addition, research on covert VTE has indicated that the effect is disrupted during auditory interference, which suggests that auditory processes are engaged during the search for VTE (Smith et al., 1995). Altogether these studies suggest that intentional inner speech monitoring can at least take place at the lower two linguistic levels, i.e., formulation and articulatory planning. Beyond these levels, it is still an open question whether inner speech monitoring may occur at the conceptualization level. Studies of self-repairs in spontaneous overt speech production show that speakers do monitor the intended pre-verbal message for appropriateness (e.g., Levelt, 1983; Blackmer and Mitton, 1991). In the overt speaking mode, monitoring seems therefore to occur during conceptualization. In children’s private speech, which, as mentioned above, has been argued to be a precursor to inner speech, self-repairs are also present at the conceptualization level, as shown by occurrences of re-wording or amending of utterances (e.g., Manfra et al., 2016). Consequently, the feedback arrows in Figure 1 represent the self-editing processes that may take place at all levels during intentional inner speech, including conceptualization. However, this monitoring may be less stringent than the one that operates in the overt mode. As mentioned above, Egger (1881), Vygotsky (1934/1986), Bergounioux (2004), or Wiley (2006a) claim that inner speech only needs to be understood by ourselves, which implies that we can be less distinct, that we can abbreviate inner sentences and that we can even sometimes produce erroneous forms, as long as meaning is preserved. Wiley (2006a, 2014) proposed that the control processes in overt and covert modes are different. In inner speech, efficiency rules prevail, so that production can be sped up and economized. Linguistic rules are therefore weakened and monitoring can be considered as more lax in intentional inner speech than overt speech. As concerns less intentional forms of inner speech, that occur during mind wandering, to our knowledge, there are no studies showing that monitoring mechanisms are at play. By definition, mind wandering operates without executive control, or with only intermittent control (but see Smallwood et al., 2012). In the present paper, we therefore assume that verbal monitoring is reduced during verbal mind wandering, hence the absence of self-editing arrows on the unintentional side in Figure 1.

### The ConDialInt Model: Functional Neuroanatomy of Multidimensional Inner Speech

**FIGURE 2:**
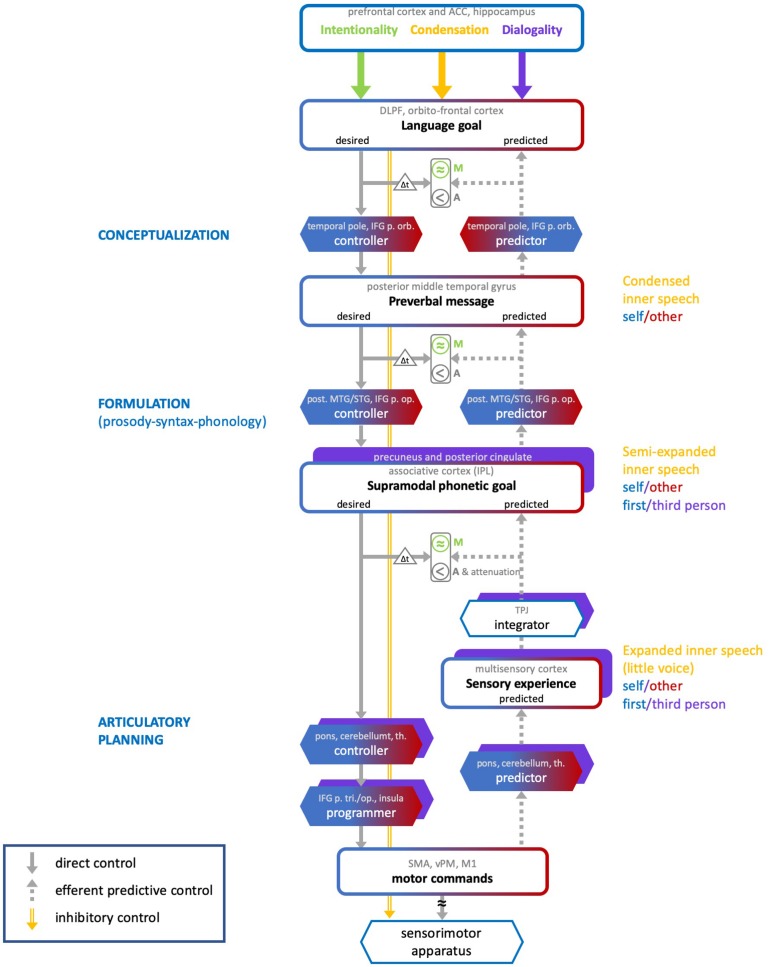
The ConDialInt neurocognitive model of multidimensional inner speech. ACC, anterior cingulate cortex; DLPF, dorsolateral prefrontal; IFG, inferior frontal gyrus; p. orb, *pars orbitalis*; p. op., *pars opercularis*; p. tri, *pars triangularis*; MTG/STG, middle temporal gyrus/superior temporal gyrus; IPL, inferior parietal lobule; TPJ, temporo-parietal junction; th., thalamus; SMA, supplementary motor area; vPM, ventral premotor cortex; M1, primary motor area.

We propose a neurocognitive model that accounts for the varieties of inner speech along the three dimensions described above, and for their monitoring. The ConDialInt model (for Condensation-Dialogality-Intentionality) is based on the preliminary account presented in Lœvenbruck et al. (2018), which focused on the latest stage of the production of intentional inner speech, i.e., articulatory planning. In this preliminary account, inner speech monitoring was based on a predictive control scheme, inspired from Frith et al. (2000) and also described in Rapin et al. (2013) and Perrone-Bertolotti et al. (2014). In Lœvenbruck (2018), a provisional extension of this account has been sketched, in which formulation and conceptualization stages were added to the articulatory planning stage. We further elaborate on these propositions and consider a more comprehensive neurocognitive model which addresses the three dimensions of inner speech (Figure 2). The ConDialInt model is limited to oral language (inner speech), since available data on inner sign language production are too scant, but we speculate that the auditory processes and representations invoked here for inner oral language may be replaced with visual elements to account for inner sign language.

In the ConDialInt model, verbal monitoring is based on a hierarchical predictive control scheme. Such a scheme has been originally proposed for complex movement control by Haruno et al. (2003) and Pacherie (2008). Predictive control has been successfully implemented in speech motor control (e.g., Postma, 2000; Guenther et al., 2006; Houde and Nagarajan, 2011). It is based on the pairing of two types of internal models, a forward model (predictor) and an inverse model (controller). The inverse model computes a motor command, while the forward model predicts the consequence of the ongoing command, using an efference copy of this command. Monitoring is based on several comparisons between desired, predicted and actual sensory outcomes. The crucial comparison involves predicted and desired signals: it allows errors to be monitored before the action is even accomplished. In hierarchical predictive control, pairs of controllers and predictors are organized in cascade, with bidirectional information processing across levels. This type of control has been applied to overt language production by Pickering and Garrod (2013). According to them, monitoring can take place at all stages of language production, using a predictive scheme: Actual and predicted semantics can be compared, as well as actual and predicted syntax, and actual and predicted phonology. Any mismatch between actual outputs and predictions may trigger a correction, by tuning the internal models at each stage. The ConDialInt model is an adaptation and extension of Pickering and Garrod’s (2013) hierarchical predictive control model of overt speech production to covert speech production. Importantly, compared with Pickering and Garrod’s original model, it provides a detailed implementation of the predictive control scheme at each of the hierarchical levels. This fine-grained implementation of predictive control enables us to describe the varieties of inner speech along the condensation dimension by integrating an inhibitory control mechanism that can be applied at different levels in the hierarchy. The higher the speech production flow is interrupted, the more condensed the inner speech variety is. It accounts for dialogality by replacing the speaker’s own internal models with internal models that simulate other speakers’ vocal productions and by including perspective switching mechanisms (from speaker to addressee). Finally, it accounts for intentionality by incorporating different degrees of production monitoring.

Another predictive account of inner speech has been provided by Wilkinson and Fernyhough (2017). Their account takes a predictive processing approach, stemming from Friston’s (2005) active inference theory. Our own model is compatible with many of their hypotheses, but slightly differs in a number of ways. First, as explained below, we claim that inner speech, in its most expanded form, does entail a stimulus, a sensation, and that this sensation is a prediction, derived from motor commands. Second, we argue that inner speaking (in an expanded way) is indeed imagining oneself speaking, i.e., simulating the act of speaking, and that this simulation can take place with different voices, giving rise to different percepts. We speculate that speakers develop internal (or generative) models of themselves as well as of others. And these internal models allow them to simulate different voices. Third, we assume that the ability to engage in dialogs (covertly and overtly) comes with a mechanism by which speakers can hold track of perspectives. This mechanism allows one to imagine that someone is speaking to them. As we describe below, it is precisely this ability which explains the move from “me speaking” to “other speaking” that Wilkinson and Fernyhough argue is lacking in more traditional self-monitoring models of inner speech. We contend that this perspective switching ability, together with voice modulation (own voice vs. other voice), lies at the origin of auditory verbal hallucination, when self-monitoring goes awry.

Our model resolves a few ambiguities in Pickering and Garrod’s original model, which does not specify in detail what the forward-inverse pairs implement at each of the hierarchical levels. In our view, at the lowest level (articulatory planning), the predictor-controller pair functions just as described in typical predictive control models of action control (e.g., Miall and Wolpert, 1996). The predictor model is thus a model of the biophysical speech apparatus, that converts motor commands (or rather efference copies of motor commands) into predicted articulatory movements and their resulting sounds and somatosensory percepts. At the higher levels (formulation and conceptualization), however, there is no biophysical apparatus to be modeled, and no movements or sounds to be predicted. The predicted representations at these levels are abstract phonetic goals and preverbal messages. We assume, therefore, that the pairs of predictors and controllers in the two highest hierarchical levels are not models of any biophysical apparatus. They are computational procedures that convert one type of mental representation (e.g., broad language goal) into another type of mental representation (e.g., preverbal message). Consequently, in the ConDialInt model, hierarchical predictive control of inner speech runs as follows. At the conceptualization stage, the broad language goal is converted into a desired preverbal message by a conceptualization controller. This desired preverbal message is the highly condensed inner speech percept. It is sent back as input to a conceptualization predictor, which predicts the language goal that would derive from it. Desired and predicted language goals can thus be compared, provided that the desired goal is buffered, so that desired and predicted signals are temporally aligned (as represented by the Δt triangle in Figure 2). Any error at this early monitoring stage can be corrected for, by sending an error signal to the conceptualization controller and by delaying lower level processes. At the formulation stage, the desired preverbal message is converted into a desired phonetic goal by a formulation controller. This desired phonetic goal corresponds to a semi-expanded inner speech percept and can be transformed (in the articulatory planning stage) into motor commands. In robotics or limb control theory, goals are desired configurational states of the peripheral motor system, specified in terms of position and velocity of the motor apparatus (e.g., Miall and Wolpert, 1996). This is appropriate for movements of the hand or arm. In the case of dynamic speech control, it is unlikely that the phonetic targets of the speakers are exclusively specified in terms of spatial configurations, i.e., positions and velocities of the speech articulators. Many studies suggest instead that speech targets are defined in both auditory and articulatory terms (for arguments on auditory targets see e.g., Perkell et al., 1997 or Guenther et al., 2006; for arguments on articulatory, i.e., somatosensory, targets, see Saltzman and Munhall, 1989, Browman and Goldstein, 1989 or Tremblay et al., 2003; for arguments on auditory-somatosensory targets, see e.g., Lœvenbruck, 1996, Patri et al., 2018, Perkell, 2012 or Perrier et al., 1996). We therefore argue that the phonetic goal is a supramodal integration of auditory and somatosensory (and perhaps even visual) representations. A formulation predictor can transform the phonetic goal back into a predicted preverbal message, which can be compared with the (buffered, see Δt triangle) desired one. Any error at this intermediate monitoring stage can be corrected for by sending an error signal to the formulation controller (and perhaps also, by bottom-up cascade, to the conceptualization controller) and by delaying lower level processes. It has been claimed that the formulation stage itself can be divided into grammatical and phonological encoding (see e.g., Levelt, 1989). In this case, then, the pair of controller-predictor at the formulation stage should be replaced with two pairs, one for each sublevel. Lastly, at the articulatory planning stage, the desired phonetic goal is converted into motor commands by an articulatory-planning controller. In the case of overt speech, the motor commands are fed to the speech apparatus, resulting in articulatory movements and sounds. In the case of covert production, the motor commands are inhibited, resulting in no movement of the speech apparatus. In both overt and covert cases, an efference copy of the motor commands is sent to an articulatory-planning predictor which generates a predicted sensory experience (ahead of the actual experience, in the case of overt speech).

This sensory experience corresponds to the percept of an inner voice, with auditory as well as somatosensory qualities. As we have argued in Lœvenbruck et al. (2018) and Perrone-Bertolotti et al. (2014), inner speech can be associated with auditory as well as somatosensory representations. Somatosensory representations include tactile and proprioceptive sensations in the speech organs, that, like auditory sensations, result from imagined articulatory gestures. The claim that the inner voice has auditory qualities is supported by introspective data on timbre, pitch, and intensity (e.g., Egger, 1881), by behavioral findings (e.g., Reisberg et al., 1989; Smith et al., 1995; Corley et al., 2011; Dell and Oppenheim, 2015) and by neuroimaging data (e.g., Bookheimer et al., 1995; Sato et al., 2004; Lœvenbruck et al., 2005; Basho et al., 2007). The assumption that somatosensory representations may sometimes also be at play comes from introspective data (Taine, 1870; Paulhan, 1886) as well as a few neuroimaging results (e.g., Rosen et al., 2000; Huang et al., 2002). Further empirical data are needed to define whether somatosensory signals are systematically involved during expanded inner speech. Our model includes this possibility. The argument that these multisensory signals result from simulated motor actions of the speech organs is itself supported by introspective experiments (Bain, 1855; Stricker, 1885), physiological measurements (Jacobson, 1931; Sokolov, 1972; Conrad and Schönle, 1979; McGuigan and Dollins, 1989; Livesay et al., 1996) as well as neuroimaging data (Bookheimer et al., 1995; McGuire et al., 1996; Baciu et al., 1999; Palmer et al., 2001; Shergill et al., 2001; Huang et al., 2002; Basho et al., 2007; Partovi et al., 2012).

The multisensory experience is integrated into a predicted supramodal representation which can be compared with the (buffered, see Δt triangle) desired phonetic goal. Any error at this last monitoring stage can be corrected for by sending an error signal to the articulatory-planning controller (this error signal may perhaps also be fed back to higher-level controllers) to issue new commands. In the case of overt speech production, this allows for errors to be corrected before the utterance is even produced, a strong argument for predictive control. In action control, it has been claimed (by Frith et al., 2000, for instance), that the efference copy mechanism is crucial to the sense of agency, the feeling of being the agent of our own action. In Rapin et al. (2013) and Lœvenbruck et al. (2018), it was argued that, in inner speech, the sense of agency is derived from the comparison between desired and predicted signals (see also Tian and Poeppel, 2012 and Swiney and Sousa, 2014). We further elaborate on this assumption, by claiming that the comparisons between desired and predicted signals at each level provide a sense of agency (referred to as “A” in Figure 2) of the inner production. This is represented with a “<” sign at each level, symbolizing the presence of a desired signal ahead of the predicted signal. Several studies have reported dampened neural response in auditory cortex during inner speech and silently mouthed speech compared with speech perception (e.g., Ford and Mathalon, 2004; Agnew et al., 2013). One interpretation is that the monitoring mechanism not only allows to check that predicted signals are similar to the desired ones, but also plays a role in sensory attenuation. When desired and predicted signals match, a dampening of the self-generated sensory experience takes place, so that any external sensory experience is easier to detect (e.g., Blakemore et al., 2002; Ford and Mathalon, 2004). The ConDialInt model therefore includes an attenuation mechanism at the articulatory planning stage, when desired and predicted signals are consistent.

As concerns the condensation dimension, the ConDialInt model includes inhibitory control mechanisms at each hierarchical level (orange arrow in Figure 2). The level at which the speech production flow is inhibited defines the degree of condensation. Inhibition at the formulation stage interrupts production at the preverbal message and results in highly condensed inner speech. Inhibition at the articulatory planning stage terminates production at the phonetic goal, giving rise to a semi-expanded variety. When inhibition occurs further down the production flow, it cancels out motor commands but a predicted sensory experience can still be computed. Therefore, inhibition at this level prevents articulatory gestures from being generated but releases the experience of expanded inner speech, with auditory and somatosensory qualities, i.e., the little voice we can hear in our head.

The ConDialInt model also accounts for dialogality. When inner speech is produced with one’s own voice, the processes described above simply unfold, stopping at various stages, depending on the condensation dimension. When one covertly imitates someone else’s voice, the controller and predictor internal models are adapted, modulated, in order to control and predict another voice than one’s own. Pickering and Garrod (2013) have claimed that their hierarchical predictive control scheme can also account for efficient speech comprehension, by deriving predictions of the interlocutor’s language goals, using predictor models. This implies that listeners are able to build adapted internal models of their interlocutor, at the different stages of language processing. Indeed, when we know someone’s voice, and know them well, we can often also recognize their phonological, lexical, syntactic, and prosodic habits. In such cases, we can therefore, presumably, make reasonably accurate adaptations of our own predictors and controllers, that fit with our interlocutors’ features, at each linguistic level. Similarly, when we covertly imitate someone, adaptations of the controller-predictor pairs at each stage could also be made, resulting in predicted signals that correspond to a different inner voice than our own. In Figure 2, the possibility of adapting predictors and controllers is represented with a blue-red fading pattern (with blue for self, and red for others). The outputs of the predictors and controllers at each stage (which correspond to inner speech varieties) are represented with blue-red bordered boxes. Moreover, dialogality (in the polyphonic sense explained above) also implies switches in perspective. Not only can we mentally imitate someone’s voice, but we can also imagine that someone else is talking to us. Dedicated neural mechanisms have been shown to be at play when participants are asked to imagine being the agent of the action or when they imagine another person being the agent (Ruby and Decety, 2001). Compared with imagining being the agent (first-person perspective), imagining another person being the agent (third-person perspective) has been shown to elicit responses in the right inferior parietal lobule, the precuneus, the posterior cingulate, and the fronto-polar cortex. In line with these findings on motor imagery, we assume that the dialogality dimension involves a perspective switching mechanism, as well as further monitoring and executive control processes. In monologal inner speech, a first-person perspective is taken, in which one imagines being the agent of the speech action. In dialogal inner speech, a third-person perspective is taken, in which one imagines another person being the agent. The perspective switch, from first-person to third-person, probably occurs during the latest stage of speech production, i.e., during articulatory planning, when physical embodiment takes place and the voice is being generated (predicted). The initial stages, conceptualization and formulation, are more abstract, less embodied, and can be initiated with one’s own or someone else’s linguistic habits. Up to these stages, imagining someone else speaking (rather that oneself) merely requires using internal models that are adapted to that individual’s linguistic characteristics (lexicon, syntax, prosody). Changing the agent of the imagined verbal action does not otherwise modify conceptualization and formulation. Articulatory planning, on the other hand, is affected by the change in agent, since it is the stage at which the verbal material becomes physically instantiated, with full articulatory specification. Articulatory planning involves predicting the temporal dynamics of the position and velocity of the speech articulators. When one imagines oneself speaking, these articulatory configurations are computed from a first-person perspective. When one imagines another individual speaking, the dynamics of the configurations of the speech apparatus is computed with a third-person perspective. The ConDialInt model therefore includes a mechanism by which this change in point of view can operate. This is illustrated in Figure 2, by the addition of purple boxes at the articulatory planning stage, which account for the perspective switch that operates in dialogal inner speech.

As concerns the intentionality dimension, we argue that verbal monitoring only concerns intentional inner speech. During intentional inner speech, the signals generated by the controllers at each level are converted by predictors into predicted signals that are issued back one level-up in the hierarchy to be compared with initial desired signals. As stressed above, the comparison process is more lenient than in overt speech, hence the approximate symbols in Figure 2. In unintentional inner speech, we assume that no verbal monitoring takes place: unbidden verbal thoughts arise, but they are not confronted to initial objectives. Therefore, the control is merely feedforward, but comparisons between predictions and goals may still take place, for agency to be felt. Even unintentional inner speech comes with a feeling of agency. When that feeling is defective, auditory verbal hallucination may occur. In the ConDialInt model a distinction is therefore made between verbal monitoring (M), which only concerns intentional varieties (represented in green in Figure 2), and agency attribution (A), which concerns all varieties.

We speculate on a tentative neuroanatomical grounding for this functional account, based on previous neuroimaging studies and descriptions. The predominantly left-lateralized neural regions associated with the different processes are listed in each box in Figure 2. As concerns the conceptualization stage, following considerations by Blank et al. (2002), Caplan et al. (2000), Duffau et al. (2014), Gernsbacher and Kaschak (2003), Haller et al. (2005), Hickok (2009), Indefrey et al. (2001), Indefrey and Levelt (2004), Lœvenbruck et al. (2005), Rauschecker and Scott (2009), Tian and Poeppel (2013), and Tremblay and Dick (2016), we assume that the ventral stream of regions engaged are predominantly left-lateralized and include the dorsolateral prefrontal cortex (DLPFC), the orbitofrontal cortex, the *pars orbitalis* of the inferior frontal gyrus, the temporal pole and the posterior middle temporal gyrus, with ventral temporo-frontal connections presumably involving the inferior occipito-frontal fasciculus (fascicles are not mentioned in Figure 2, for simplification).

Next, based on consideration by Duffau et al. (2014), among others, we presume that the formulation stage, which generates lexico-prosodico-syntactico-morpho-phonological representations, involves a dorsal stream, with recruitment of the posterior part of the left superior and middle temporal lobe as well as the left inferior frontal gyrus (IFG, *pars opercularis*) and with dorsal connections via the superior longitudinal fasciculus, as well as the arcuate fasciculus. We add that the left inferior parietal lobule (IPL) is recruited at this stage, to form the supramodal phonetic goal. We have argued that the phonetic goal is in an integrated supramodal format, which is consistent with IPL recruitment. But it is still an open question whether, at this formulation stage, the activation of the left IFG precedes that of the IPL or whether, instead, the IPL itself provides efferences to the IFG. Figure 2 opts for the first scheme (at the formulation stage).

**FIGURE 3:**
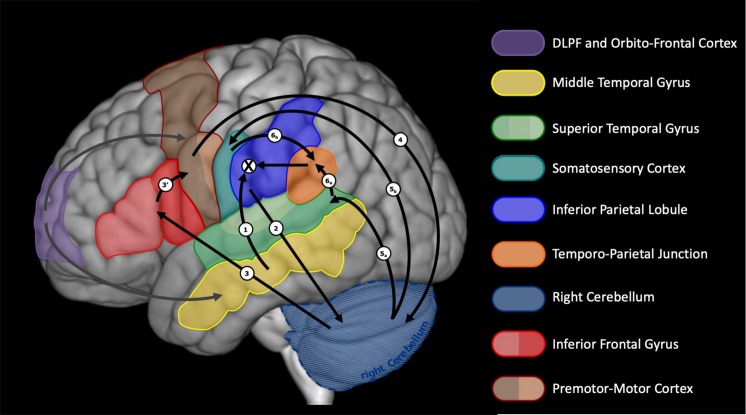
Neuroanatomical network of articulatory planning during expanded inner speech. A tentative description of the sequences of activation is provided, ending up with the comparison between desired and predicted phonetic goals within the inferior parietal lobule. Numbers refer to the assumed sequence of activations. The cross sign refers to the comparison that takes place between the intended phonetic goal and the integrated multisensory prediction.

We claim that, for expanded varieties of inner speech, articulatory planning follows. A preliminary neural network for this last stage was presented in Lœvenbruck et al. (2018). This proposition was based on considerations and models by Indefrey (2011), Guenther and Vladusich (2012), Hickok (2012), and Tian and Poeppel (2013), among others. We slightly revise this initial proposition to better capture the notion of supramodal phonetic goal described above, to allow for suggestions by Flinker et al. (2015) and by Duffau et al. (2014) on temporo-frontal connections, and to include recent considerations on the role of the cerebellum in language production and internal models (see e.g., Imamizu and Kawato, 2009; Buckner et al., 2011; Smet et al., 2013; Mariën et al., 2014; Diedrichsen and Zotow, 2015; Sokolov et al., 2017). Our speculation takes advantage of the double representation of cerebral regions in the anterior and posterior lobes of the cerebellum (see e.g., Sokolov et al., 2017). Figure 3 illustrates this revised view of the left cerebral and right cerebellar regions involved. The phonetic goal is sent from the left inferior parietal lobule (or the left IFG, if IPL-IFG connections are in the reversed order, see above) to the cerebellum (possibly the anterior lobe), via the pons. A conversion takes place through the controller in the cerebellum, which generates a motor specification sent to the left frontal regions via the thalamus. Motor programs are then issued, by coordinating the motor specification, stemming from the cerebellum, with ongoing speech actions. We speculate that the regions involved in this process are the triangular and opercular IFG and the anterior insula, then the ventral premotor cortex, the supplementary area and the primary motor cortex (via the frontal aslant track, not shown in Figures 2, 3). There are arguments for the hypothesis that the IFG recruitment precedes ventral premotor cortex activation (e.g., the electrocorticography speech production study by Flinker et al., 2015) and that the inferior parietal lobule (supramarginal gyrus) efferences toward the ventral premotor cortex, via the anterior part of the superior longitudinal fascicle (Duffau et al., 2014). There are also arguments for the existence of connections from the IPL toward the cerebellum (Miall, 2003; Imamizu and Kawato, 2009) and from the cerebellum to the frontal motor and premotor areas, possibly including the IFG (Imamizu and Kawato, 2009; Murdoch, 2010). What remains unclear, is whether the direct (not mediated by the cerebellum) parieto-frontal connection is associated with the articulatory planning stage or only relevant to the formulation stage (as assumed here). We claim that the motor commands that result from the motor specification are not issued to the speech apparatus (inhibition) but they are sent, via the pons, to the cerebellum (possibly the posterior lobe), which, we speculate, includes a predictor. We further speculate that the cerebellum issues, via the thalamus, a multisensory prediction, which is processed by the auditory cortex (superior temporal gyrus) and the somatosensory cortex (postcentral gyrus). This multisensory prediction gives rise to the percept of an inner voice, that unfolds over time. The sequence of activation from inferior parietal to temporal cortex (mediated, we argue, by cerebellum and inferior frontal regions) is compatible with the MEG data obtained by Tian and Poeppel (2010). In an articulation imagery tasks, they found that the auditory response was elicited around 170 ms after a posterior parietal activity (where we think the phonetic goal is built) was recorded. We speculate that the auditory and somatosensory responses are further integrated into a supramodal representation, via the temporo-parietal junction (TPJ). The resulting supramodal phonetic prediction is compared with the desired phonetic goal within the IPL and monitoring can take place. Note that in this account, the IFG is involved at two stages. In an early stage, during formulation, we consider that the triangular part of the IFG plays a role in the monitoring of thematic roles (who-does-what-to-whom) that is crucial to morphosyntactic processing (see Caplan and Hanna, 1998; Caplan et al., 2000; Indefrey et al., 2001; Lœvenbruck et al., 2005). In a later (articulatory planning) stage, we claim that the opercular part may be involved in the coordination and sequencing of articulatory gestures (Blank et al., 2002; Indefrey and Levelt, 2004).

Moreover, we presume that cognitive control, which has been defined as the “ability to orchestrate thought and action in accordance with internal goals” (Miller and Cohen, 2001) must take place to inhibit motor execution and to interrupt production before articulatory planning, when appropriate (condensation dimension). Cognitive control is also needed to launch the adaptation of internal models (controllers/predictors) at each stage, when different voices are imagined (dialogality dimension), and to tune the strength of the monitoring processes depending on the degree of willfulness (intentionality dimension). Cognitive control has been shown to recruit various regions of the prefrontal cortex (PFC), including dorsolateral PFC, ventrolateral PFC, orbitofrontal cortex, and anterior cingulate. It is still debatable what the roles of the different subregions of PFC are and it is beyond the purpose of this paper to describe them. We refer to Ridderinkhof et al. (2004) for more detail. We have therefore added the prefrontal cortex and the anterior cingulate cortex (ACC) above all processes. In addition, the modulation and adaptation of internal models during dialogal inner speech presumably requires memory retrieval processes, in search of the voice quality and linguistic features of the imagined other. We have therefore added the hippocampus in the set of crucial regions. Furthermore, as mentioned above, the right IPL, the precuneus, the posterior cingulate, and the fronto-polar cortex are claimed to play a role in first-/third-person perspective taking (Ruby and Decety, 2001; Decety, 2005). Decety and Grèzes (2006) provide further argument for the role of the right IPL in the attribution of actions, emotions, and thoughts to their respective agents when one mentally simulates actions for oneself or for another individual. Their review of the literature show that it is difficult to assess whether the crucial region in this process is the rostral part of the right IPL or the right TPJ. The purple boxes in Figure 2 for the operations of phonetic goal construction, sensory experience processing and multisensory integration, represent the perspective switching operations, which presumably include a shift in hemispheric dominance, from left to right IPL and/or TPJ, as well as recruitment of the precuneus and posterior cingulate.

### Assessing the Neural Networks Mediating Multidimensional Inner Speech

The aim of the present study is to examine the neuroanatomical assumptions of the ConDialInt model by investigating the neural correlates of multidimensional inner speech using fMRI. Previous fMRI studies of inner speech did not address dialogality and intentionality simultaneously.

Along the dialogality dimension, the study by Tian et al. (2016) compared inner speaking (articulation imagery) and imagining someone else speaking (hearing imagery), but only single syllables were used, which is restrictive. In addition, the participants were explicitly trained to mentally articulate during inner speaking, while they were asked to minimize articulatory feeling and rely instead on auditory memory processes during hearing imagery. These results are interesting but they are not sufficiently informative as to which neural networks are involved in less constrained inner speech (i.e., during full sentence production and with less attentional focus on articulatory sensation and auditory memory). The study by Alderson-Day et al. (2016) addressed dialogality in a more ecological way, using scenarios designed to elicit either monologal (soliloquial) or dialogal (imagining a dialog with another person) inner speech. Participants used one single voice in the monologal condition and several voices in the dialogal condition. Therefore, comparing these two conditions does not allow to conclude on the processes that specifically underlie perspective shifting, without the confounding factor of voice modulation.

Along the intentionality dimension, Hurlburt et al. (2016) carefully addressed the difference between intentional monologal and unintentional monologal inner speech (which they refer to as spontaneous inner speaking). They also investigated unintentional dialogal inner speech (referred to as spontaneous inner hearing). Although unintentional monologal inner speech was relatively frequent, occurring in 29 percent of their samples and for each of their five participants, unintentional dialogal inner speech was rare (occurred zero times or twice) for three participants. Further data are therefore needed on dialogal inner speech.

The conditions in the present study were specifically designed to compare inner speech varieties along the two dimensions of dialogality and intentionality. To explore dialogality, three controlled inner speech conditions were compared, during which participants were instructed to mentally generate verbal definitions of visually presented words (they were primed with a written word and its pictorial illustration). In the intentional monologal self-voice condition, participants were asked to covertly produce a definition, with their own voice. In the intentional dialogal other-voice condition, they were instructed to imagine that someone was producing an utterance addressed to them. Compared with the monologal self-voice condition, this condition requires two additional processes: mentally altering one’s voice, which implies prosodic and voice quality control, and taking an allocentric perspective. To specifically examine perspective taking, without the confounding factor of voice alteration control, we added an intermediate condition in which participants were asked to covertly produce a definition, with someone else’s voice (intentional monologal other-voice). To explore the intentionality dimension, in addition to these conditions, a mind wandering session took place, after which participants were asked to report any spontaneously occurring verbal material. The mind wandering session was also meant to allow us to explore the condensation dimension. To assess to what extent auditory processes are at play during inner speech, we added a speech perception condition.

## Materials and Methods

### Participants

Twenty-four healthy native speakers of French were included (10 men; mean age = 29.5 years, SD = 10.04; 14 women, mean age = 28.07 years, SD = 8.14). All participants were right-handed (Edinburgh Handedness Inventory; Oldfield, 1971), scored average on a mental imagery questionnaire (based on Sheehan, 1967), had normal or corrected-to-normal vision and had no history of neurological or language disorders. Each participant gave written informed consent and received 30€ for their participation. Ethical approval was granted by the Comité de Protection des Personnes (CPP) Sud-Est V and by the National Competent Authority France-ANSM (Ref. CPP: 14-CHUG-39, Ref. Promoteur: 38RC14.304, ID-RCB: 2014-A01403-44, Ref. ANSM: 141200B-31, ClinicalTrials.gov ID: NCT02830100).

### Tasks

Participants were first introduced to an avatar, who gave them instructions and provided training for the five conditions. The avatar had a saliently high-pitched voice which was sufficiently strange (outside of an adult’s typical pitch range), yet easy to imitate for everyone. The first four conditions included one speech perception condition and three intentional inner speech conditions. In these four conditions, each trial started with the visual presentation of a written word and its illustration. For example, the written word “ball,” with a picture of a ball (framed within a stylized clock) was visually presented for 2 s, after which the clock rotated and the participant performed the task, which lasted for 4 s. Each trial was repeated several times in each condition (see section “Stimuli”). In the “Speech Perception” (SP) condition, participants had to listen to the definitions presented to them via MR compatible earphones. The definitions were pronounced by the avatar with the high-pitched voice. Each definition began with “This is something…”. In the Monologal Self-voice inner speech (MS) condition, participants had to mentally generate definitions of each of the visually presented objects, using a sentence beginning with “This is something.” Participants were not reading sentences, they had to generate their own definitions. The stimuli were purely visual (no audio presentation of the word). The Monologal Other-voice inner speech (MO) condition was similar to the MS condition, except that participants had to mentally imitate the high-pitched voice of the avatar. In the Dialogal Other-voice (DO) condition, participants had to imagine that the avatar was addressing them, producing a sentence starting with “Here is a typical image of a…” and ending with the name of the object, without generating a definition (to reduce cognitive load). The fifth condition investigated “Verbal Mind Wandering” (VMW). In this condition, a written word and its illustration was first visually presented for 2 s, in order to provide the same initial visual stimulation as in the other four conditions. After the initial 2 s written word-illustration presentation, participants were asked to fixate a stylized clock rotating for 30 s. They were instructed to monitor spontaneously occurring thoughts. At the end of the trial, they reported the periods during which they experienced verbal thoughts, by selecting time portions on the stylized clock which appeared on the screen, using a joystick. The stimulus presentation and collection of joystick responses were controlled using the Presentation software (Neurobehavioral systems)^1^.

### Stimuli

Four 30-word lists of nouns were created using the LEXIQUE database (New et al., 2001). In order to facilitate the generation of definitions, only frequent and imageable words were chosen. All nouns were of neutral affective content and included the categories of food, houseware, furniture, clothing and transportation devices. Each list was randomly assigned to one of the first four conditions. The lists were the same for all participants. They were carefully matched for syllable counts, frequency, familiarity, concreteness and imageability. Only one item was presented (a clock) in the fifth condition (VMW).

The audio stimuli (for the SP condition) and the instructions were recorded by two female native speakers of French in a quiet room. One speaker generated the avatar’s voice contents, i.e., tasks instructions for SP, MO and DO, as well as definitions used in the SP condition. The other speaker generated instructions for the remaining conditions (VMW and MS). Audio signals were digitized with a sampling frequency of 44199 Hz and 32-bit resolution, then normalized in amplitude to the mean power of all stimuli. The recorded definitions in the 30 test trials for the SP condition lasted on average 2.87 s (SD = 0.44).

### Expected Outcomes

Comparing the monologal self-voice (MS) condition with baseline should help assessing the predictive control hypothesis. Namely, it is expected that expanded inner speech in the MS condition should recruit speech production processes down to articulatory planning, resulting in a predicted signal, the inner voice, with auditory qualities. It is expected that compared with baseline, MS should recruit hippocampus and posterior middle temporal gyrus for the conceptualization stage. The posterior temporal lobe and left inferior frontal gyrus should be recruited for the formulation stage. The left inferior parietal lobule should be activated for the articulatory planning stage (for the specification of the supramodal phonetic goal), as well as the right cerebellum (controller model, for motor commands specification and predictor model for sensory prediction), the left premotor cortex, left IFG and insula (for motor command coordination) and the auditory cortex (for sensory processing). Somatosensory cortex might also be recruited. Furthermore, the prefrontal cortex (middle and superior frontal regions) should be recruited to issue inhibitory control signals, preventing movement of the speech apparatus.

Comparing the MS condition with the speech perception (SP) condition should further assess whether auditory processing is at play during expanded inner speech and whether some attenuation occurs, relative to actual speech perception, as predicted by the model.

Comparing monologal other-voice (MO) and dialogal other-voice (DO) each with the baseline and with SP should further test the predictive control hypothesis and assess the recruitment of motor and auditory processes. Comparing MO with MS should shed light on the first aspect of dialogality, namely voice modulation. Given that the most striking feature of the voice to be mentally imitated was its high pitch, it can be speculated that in MO, intonation control regions should be recruited. In particular, it can be expected that the right inferior frontal gyrus should be activated. In addition, the internal models used in MS (and presumably associated with right cerebellar activation) should be replaced with internal models adapted to this new voice. The cerebellar recruitment might therefore differ in these two conditions.

Comparing DO with MO should shed light on the second aspect of dialogality, namely perspective shifting. Based on Ruby and Decety’s (2001) study on perspective shifting, it can be expected that, relative to MO, DO should additionally activate the right parietal cortex, and more specifically, the inferior and superior parietal lobules as well as the precuneus and the posterior cingulate.

Comparing the verbal mind wandering (VMW) condition to the baseline should contribute to better describe the intentionality dimension and could potentially shed light on the condensation dimension. It can be expected that compared with the baseline, VMW should activate the default mode network as well as speech production regions. Comparing VMW and MS, MO and DO could potentially provide insight on the neuroanatomical differences between varieties of inner speech along the intentionality dimension.

### fMRI Protocol

**FIGURE 4:**
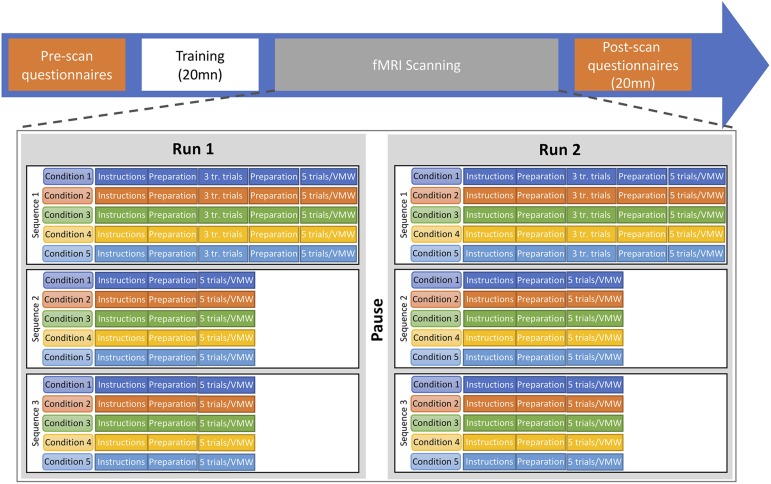
Timeline of the experimental procedure. Two functional runs were completed, each including the five conditions. Each run included three repetitions of the sequence of five conditions. In each repetition, five trials of the MS, MO, DO, and SP conditions were elicited as well as one VMW session. tr. trials, training trials.

A repeated-block design paradigm was used, with two runs, each including all conditions (see Figure 4). In all five conditions, participants were asked to remain perfectly still, not to make any head movement and not to articulate. They were trained to do so before entering the scanner. Each run consisted of a sequence of blocks for the five conditions (e.g., SP, MS, MO, DO, VMW) which was repeated three times. Each sequence contained five trials of each of the five conditions. Thus, in each run, each condition was presented in three different blocks of five trials, resulting in 15 trials for each condition. In the SP, MS, MO, and DO conditions, trials were separated by a fixation cross displayed for 2 s. At the beginning of each block, an instruction screen was displayed for 6 s while a recording of the instructions was played in the earphones. Then five trials of the same condition were run. A fixation cross was displayed for 8 s before and after each block. When a participant was doing a task for the first time in the run, the block started with three training trials. The sequence of conditions was pseudo-randomized across participants, with DO always after MO, to reduce confusion between tasks. For each participant, the same sequence order was used for all six repetitions of sequences. This resulted in 30 test trials (two runs, three blocks of five trials in each run) plus six training trials (two runs, three training trials in each run) per condition per participant (i.e., a total of 144 trials for the first four conditions).

### Pre- and Post-experiment Questionnaires

One day before the experiment, participants filled in the Edinburgh Handedness Inventory (Oldfield, 1971) and a mental imagery questionnaire, based on and translated from Sheehan (1967). On the day of the experiment, before entering the scanner, they were trained to report on inner speech and to intentionally produce different varieties of inner speech, without articulating. After the experiment, they filled in a recall questionnaire with a list of 60 words, for which they checked whether they had generated a definition in the scanner (20 words were distractors). This aimed at testing their attention during the tasks: if participants were focused on defining the words presented to them during the intentional inner speech tasks in the scanner, when presented with those words after the experiment, they should remember finding a definition for them. Participants also filled in subjective questionnaires to report how well they performed the tasks and to describe their thought contents during VMW.

### fMRI Acquisition

Experiments were performed using a whole-body 3T MR Philips imager (Achieva 3.0T TX Philips, Philips Medical Systems, Best, NetherLands) with a 32-channel head coil at IRMaGe MRI facility (Grenoble, France). The manufacturer-provided gradient echo planar imaging sequence (FEEPI) was used. Forty-two adjacent axial slices parallel to the bi-commissural plane were acquired in non-interleaved mode. Slice thickness was 3 mm. The in-plane voxel size was 3 × 3 mm (240 × 240 mm field of view with a 80 × 80 pixel data matrix). The main sequence parameters were: TR = 2.5 s, TE = 30 ms, flip angle = 82°. Two fMRI runs were conducted while subjects performed the tasks. During the break between the two runs, a T1-weighted high-resolution 3D anatomical volume was acquired, with a 3D T1 TFE sequence (field of view = 256 × 224 × 175 mm; resolution: 0.89 × 0.89 × 1.37 mm; acquisition matrix: 192 × 137 × 128 pixels; reconstruction matrix: 288 × 288 × 128 pixels). Participants’ gazes were monitored with an eyetracker to ensure they followed instructions.

### fMRI Data Analysis

Image preprocessing and analyses were completed using SPM12 (SPM12^2^, Wellcome Institute of Cognitive Neurology, London, United Kingdom). Standard preprocessing steps were implemented, including slice time correction, rigid body motion correction, a high-pass filter at 1/512 Hz to filter low-frequency non-linear drifts, coregistration of the functional images to each subject’s T1 anatomical images, and normalization to the Montreal Neurological Institute (MNI) template. All normalized functional images were smoothed using a Gaussian filter with a full width at half maximum of 8 mm. Individual subject analyses were conducted by constructing a general linear model for each condition. Five regressors were defined: SP, MS, MO, DO, and VMW. For all conditions, regressors were modelled as box-car functions convolved with a canonical hemodynamic response function (Friston et al., 1994). Inspection of the movement parameters derived from realignment corrections suggests that head movement was limited. Movement parameters were still included as factors of no interest. The run number was added as an additional factor. For the first-level analysis, five contrasts corresponding to each regressor of interest vs. implicit baseline were computed. For the second level, several analyses have been carried out: (i) one-sample *T*-tests, in order to measure main effects of experimental conditions, (ii) conjunction analyses between each inner speech condition and SP, between all five conditions, between all four inner speech conditions, and between all inner speech conditions grouped together and SP, in order to examine whether perception processes were recruited in all varieties of inner speech, and (iii) one-way within-subject ANOVA, in order to measure differential effects between conditions (Friston et al., 2005; Henson and Penny, 2005). To study the varieties of intentional inner speech along the dialogality dimension, MS was compared with MO (effect of changing voice) and MO was compared with DO (switching from monolog to dialog). To explore the intentionality dimension, activations in the VMW condition were compared with activations in the intentional MS condition. In all analyses (except for the contrasts between MS and MO), significant voxel clusters on each *t*-map were identified with Family Wise Error (FWE) correction at *p* < 0.05. For the MS > MO and MO > MS contrasts, no activation was found at a FWE-corrected threshold. This was not completely unexpected, given that these two conditions are very similar and they only subtly differ in the quality of the voice to be mentally produced. Although this is statistically fragile, we report the results at an uncorrected threshold (*p* < 0.001), since these contrasts are interesting in the framework of our model. Moreover, these preliminary results might guide future neuroimaging studies on inner speech production and imitation, and might help identifying regions of interest. Location of cluster maxima was determined using Automated Anatomical Labeling (AAL) map (Tzourio-Mazoyer et al., 2002). In order to quantify potential hemispheric asymmetry changes between conditions (from MS to MO and DO), percent MR signal intensity variations, or percent signal changes (%SC), were extracted within a set of regions of interest (ROIs). These ROIs included Frontal Inferior Opercularis, Frontal Inferior Triangularis, Frontal Inferior Orbitalis, Precentral gyrus, Supplementary Motor Area, Superior Temporal, Middle Temporal, Supramarginal gyrus, Inferior Parietal lobule and Superior Parietal lobule, which are among the crucial regions expected to be recruited during expanded inner speech production, according to the ConDialInt model. The ROIs were anatomically defined using the AAL atlas, in both left and right hemispheres.

## Results

### Behavioral Data

For the recall task carried out after the fMRI experiment, the mean accuracy scores across subjects was 84.42% ± 16.63. Only one participant performed poorly (below 50% accuracy). This high mean score, together with the eyetracker monitoring, suggest that participants were focused on the tasks.

After each VMW trial, participants used a joystick to report the presence of verbal episodes on the stylized clock displayed on the screen. Over the two runs (six VMW trials), participants reported between 4 and 22 verbal episodes, with a mean of 13 episodes. The proportion of time spent on verbal thought in all VWM trials ranged from 4 to 67%, with a mean of 35.6% (SD = 15.04).

**FIGURE 5:**
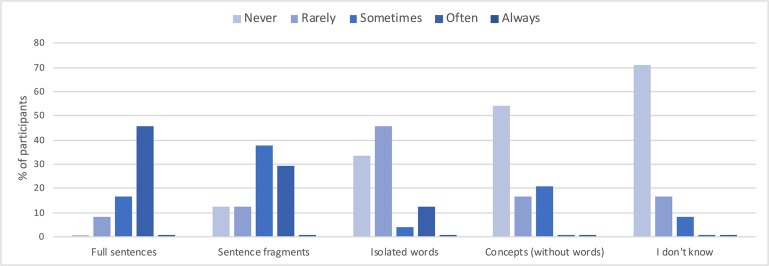
Reported degree of condensation for the inner speech episodes in the VMW condition, across all participants.

The subjective post-scan questionnaires also confirmed that the VMW condition contained verbal episodes. More specifically, concerning the condensation dimension, as the graph across all participants presented in Figure 5 suggests, the VMW condition included various degrees of condensation, from fully expanded sentences (reported as “sometimes present” in 17% of the participants and “often present” in 46%) to speech fragments (reported as “sometimes present” in 38% and “often present” in 29%), words (“sometimes present” in 4% and “often present” in 13%) and even semantic concepts without words (“sometimes present” in 21%).

In addition, the post-scan questionnaires indicate that participants rated their overall performance as correct. The MS condition was rated as easier than the MO condition, itself easier than the DO condition.

### Functional MRI Data

#### Effects of Conditions: Cerebral Correlates of Speech Perception and Inner Speech Varieties

Contrasts between each condition and the baseline are presented in Table 1, all *p* < 0.05, FWE correction. All contrasts revealed activation of the right middle and superior occipital cortex and inferior temporal (fusiform) gyrus.

**TABLE 1:** Contrasts between each condition and the baseline.

**Contrast**				**MNI** **coordinates**		
				
	**Region label**	**Extent**	***t*-value**	***x***	***y***	***z***
SP > Baseline	**Temporal_Sup_L**	**784**	**15.27**	**−63**	**−22**	**5**
	Temporal_Sup_L		11.03	–45	–22	5
	**Temporal_Sup_R**	**491**	**13.32**	**63**	**−10**	**−1**
	Temporal_Sup_R		12.90	63	–28	8
	**Frontal_Inf_Tri_L**	**345**	**8.85**	**−51**	**35**	**14**
	Frontal_Inf_Orb_2_L		8.40	–45	23	–7
	**Occipital_Mid_R**	**12**	**8.14**	**39**	**−82**	**14**
	**Precentral_L**	**27**	**7.69**	**−51**	**−7**	**47**
	**Temporal_Inf_R**	**17**	**7.51**	**45**	**−61**	**−7**
	**Supp_Motor_Area_L**	**25**	**7.26**	**−9**	**8**	**62**
	**Lingual_L**	**12**	**7.25**	**0**	**−79**	**−4**
	**Frontal_Sup_2_L**	**29**	**7.22**	**−12**	**29**	**50**
	Supp_Motor_Area_L		6.43	–6	17	56
	**Precentral_R**	**4**	**6.91**	**54**	**2**	**44**
	**Temporal_Inf_L**	**9**	**6.80**	**−45**	**−43**	**−13**
	**Hippocampus_L**	**20**	**6.74**	**−21**	**−16**	**−19**
	**Frontal_Sup_Medial_L**	**5**	**6.58**	**−9**	**47**	**41**
	**Fusiform_L**	**2**	**6.13**	**−33**	**−46**	**−19**
	**Precentral_L**	**1**	**5.99**	**−42**	**2**	**53**
MS > Baseline	**Frontal_Inf_Oper_L**	**2113**	**14.12**	**−48**	**11**	**5**
	Frontal_Inf_Tri_L		14.08	–36	26	–1
	Putamen_L		11.66	−18	11	−1
	**Frontal_Sup_Medial_L**	**771**	**12.84**	**−3**	**26**	**41**
	Supp_Motor_Area_L		11.33	–6	17	62
	Supp_Motor_Area_R		10.13	6	8	62
	**Occipital_Mid_R**	**116**	**9.97**	**36**	**−82**	**14**
	Occipital_Sup_R		8.07	18	–94	20
	**Frontal_Mid_2_L**	**37**	**7.75**	**−30**	**53**	**14**
	**Temporal_Mid_L**	**38**	**7.73**	**−51**	**−40**	**2**
	**Frontal_Sup_2_L**	**25**	**7.22**	**−9**	**53**	**35**
	Frontal_Sup_Medial_L		6.26	–9	44	41
	**Occipital_Mid_L**	**50**	**6.89**	**−39**	**−67**	**−1**
	Temporal_Inf_L		6.45	–45	–52	–16
	**Calcarine_L**	**21**	**6.81**	**0**	**−82**	**−4**
	**Precentral_R**	**5**	**6.79**	**54**	**2**	**44**
	**SupraMarginal_L**	**2**	**6.58**	**−45**	**−43**	**32**
	**Cerebellum_6_R**	**14**	**6.50**	**36**	**−64**	**−25**
	**Fusiform_L**	**5**	**6.34**	**−30**	**−46**	**−19**
	**Temporal_Pole_Sup_R**	**4**	**6.12**	**54**	**14**	**−4**
	**Hippocampus_L**	**1**	**6.00**	**−18**	**−40**	**14**
	**Insula_R**	**1**	**6.00**	**39**	**17**	**2**
MO > Baseline	**Supp_Motor_Area_L**	**661**	**11.52**	**−9**	**17**	**47**
	Supp_Motor_Area_L		10.28	–9	5	62
	**Frontal_Inf_Orb_2_L**	**717**	**11.51**	**−45**	**20**	**−7**
	Frontal_Inf_Oper_L		11.12	−51	11	5
	**Occipital_Mid_R**	**29**	**9.74**	**30**	**−85**	**17**
	**Putamen_L**	**93**	**8.54**	**−18**	**11**	**2**
	**Precentral_L**	**78**	**8.49**	**−48**	**−4**	**50**
	**Hippocampus_L**	**25**	**7.84**	**−15**	**−16**	**−19**
	**Precentral_R**	**12**	**7.67**	**54**	**−1**	**44**
	**Frontal_Mid_2_L**	**11**	**7.25**	**−30**	**50**	**11**
	**Insula_R**	**77**	**7.20**	**36**	**17**	**2**
	**Putamen_R**	**16**	**7.07**	**24**	**5**	**2**
	**Caudate_R**	**3**	**6.90**	**18**	**23**	**5**
	**Temporal_Inf_R**	**9**	**6.83**	**48**	**−67**	**−28**
	**Cerebellum_6_R**	**6**	**6.52**	**36**	**−58**	**−28**
	**Precentral_R**	**1**	**6.00**	**63**	**8**	**17**
DO > Baseline	**Occipital_Mid_R**	**230**	**10.86**	**33**	**−82**	**11**
	Cuneus_R		9	15	–94	20
	Temporal_Mid_R		8.71	48	–70	2
	**Supp_Motor_Area_L**	**503**	**10.54**	**0**	**11**	**59**
	Supp_Motor_Area_L		10.52	–6	2	65
	**Frontal_Inf_Tri_L**	**432**	**10**	**−42**	**32**	**20**
	Frontal_Inf_Oper_L		9.84	–51	11	2
	Frontal_Inf_Orb_2_L		9.78	–42	20	−7
	**Precentral_L**	**64**	**8.51**	**−48**	**−7**	**47**
	**Precentral_R**	**29**	**8.31**	**54**	**2**	**44**
	**Insula_L**	**36**	**7.53**	**48**	**8**	**−1**
	**Lingual_L**	**13**	**7.16**	**0**	**−79**	**−7**
	**Postcentral_L**	**14**	**7.01**	**−60**	**2**	**20**
	**Occipital_Mid_L**	**18**	**6.76**	**−39**	**−70**	**2**
	**Rolandic_Oper_R**	**4**	**6.54**	**60**	**8**	**14**
	**Frontal_Mid_2_L**	**1**	**6.25**	**−36**	**50**	**23**
	**Occipital_Sup_L**	**1**	**5.99**	**−9**	**−97**	**8**
VMW > Baseline	**Parietal_Sup_R**	**161**	**10.91**	**21**	**−58**	**56**
	**Frontal_Sup_Medial_L**	**305**	**10.90**	**−6**	**29**	**35**
	Supp_Motor_Area_L		7.41	−9	14	56
	Frontal_Sup_2_L		6.89	−18	17	65
	**Frontal_Mid_2_L**	**186**	**10.13**	**−30**	**50**	**14**
	Frontal_Sup_2_L		7.97	−24	44	35
	**Parietal_Inf_R**	**97**	**8.86**	**42**	**−37**	**47**
	**Temporal_Inf_R**	**107**	**8.82**	**51**	**−64**	**−4**
	Occipital_Mid_R		8.58	36	−82	17
	**Parietal_Sup_L**	**37**	**8.29**	**−18**	**−67**	**59**
	**Parietal_Inf_L**	**100**	**8.20**	**−51**	**−55**	**41**
	**Frontal_Inf_Oper_R**	**111**	**7.97**	**57**	**17**	**5**
	Insula_R		7.80	36	14	–1
	**Frontal_Inf_Orb_2_R**		6.26	48	20	−7
	**Frontal_Mid_2_R**	**66**	**7.90**	**30**	**50**	**26**
	**Supp_Motor_Area_R**	**20**	**7.26**	**15**	**20**	**62**
	**Frontal_Inf_Orb_2_L**	**148**	**7.20**	**−42**	**17**	**−7**
	Insula_L		7.17	–33	17	2
	**Occipital_Mid_L**	**4**	**6.82**	**−36**	**−73**	**5**
	**Cerebellum_Crus1_L**	**2**	**6.40**	**−33**	**−58**	**−34**
	**Frontal_Sup_2_R**	**1**	**6.10**	**24**	**14**	**65**
	**Frontal_Inf_Tri_R**	**1**	**6.07**	**48**	**35**	**−1**
	**Frontal_Sup_2_R**	**1**	**5.97**	**27**	**47**	**11**
	**Frontal_Mid_2_R**	**2**	**5.97**	**33**	**50**	**14**

*Multiple peaks in each cluster are presented at *p* < 0.05 FWE correction. Main clusters are represented in bold font, with their extent size provided. Sub-clusters are represented in regular font.*

**FIGURE 6:**
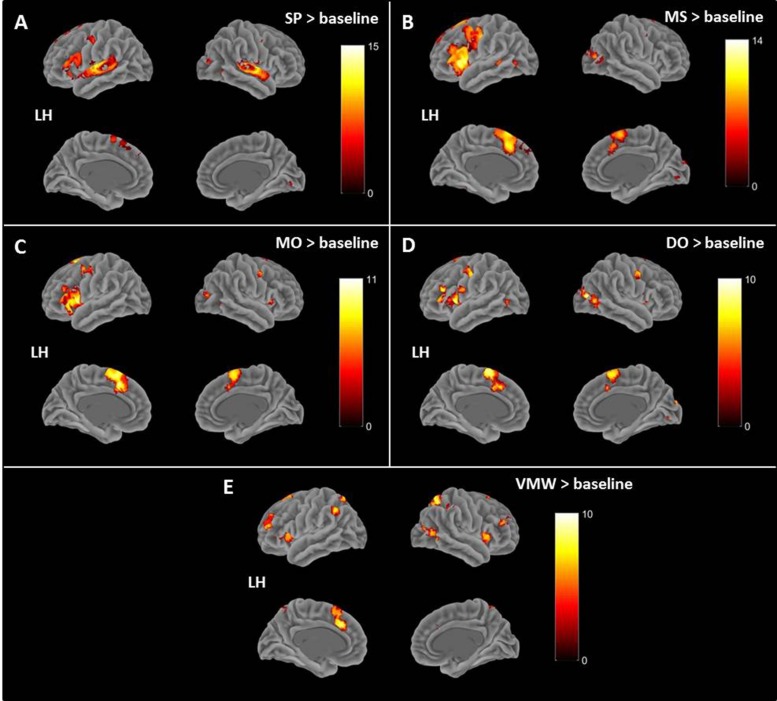
Contrasts between each condition and the baseline rendered on a standard 3D brain provided by BSPMview (Spunt, 2016). **(A)** SP > baseline. **(B)** MS > baseline. **(C)** MO > baseline. **(D)** DO > baseline. **(E)** VMW > baseline. All *p* < 0.05, FWE correction. LH, Left Hemisphere.

In addition to the activation in visual cortex, the contrast between speech perception (SP) and baseline revealed increased activation in bilateral superior temporal gyri (STG, Brodmann Area (BA) 21, 22, 41), left supramarginal gyrus (SMG, BA 40), left inferior frontal gyrus (IFG, BA 44, 47), left superior frontal gyrus (SFG, BA 8), bilateral premotor (PM) cortex, left supplementary motor area (SMA), left motor cortex, left hippocampus (Figure 6A).

Compared with baseline, intentional monologal self-voice inner speech (MS) yielded greater left hemisphere activation in the IFG (BA 44, 45, 47), middle frontal gyrus (MFG, BA 10), SFG (BA 8), SMG (BA 39), posterior middle/superior temporal gyrus (MTG/STG, BA 21, 22), hippocampus, together with bilateral SMA, bilateral PM cortex, and right cerebellum (Figure 6B).

Compared with baseline, intentional monologal other-voice inner speech (MO) revealed greater left hemisphere activation in IFG (BA 44, 47), MFG (BA 10), hippocampus, together with bilateral PM cortex, bilateral SMA, right insula (BA 13) and right cerebellum (Figure 6C).

Compared with baseline, intentional dialogal other-voice inner speech (DO) yielded greater left hemisphere activation in MFG (BA 10), middle occipital gyrus (BA 19), left insula (BA 13), together with bilateral PM cortex, IFG (BA 44, 47), and SMA (Figure 6D).

Compared with baseline, verbal mind wandering (VMW) yielded greater left hemisphere activation in SMA, together with bilateral IFG (BA 45, 47), insula (BA 13), MFG (BA 9, 10), SMA, medial SFG (BA 9), inferior (BA 39) and superior (BA 7) parietal cortex, precuneus, and left caudate, thalamus, and cerebellum (Figure 6E).

#### Common Neural Correlates for Inner Speech and Speech Perception

To investigate whether perception processes were recruited in all varieties of inner speech, conjunctions between SP and either MS, MO, DO, or VMW were examined. Conjunctions between each condition and SP are presented in Table 2, all *p* < 0.05, FWE correction.

**TABLE 2:** Conjunction analyses.

**Conjunction**				**MNI** **coordinates**		
				
	**Region label**	**Extent**	***t*-value**	***x***	***y***	***z***
**Conjunction between each of the four inner speech conditions and SP**						
MS and SP	**Occipital_Mid_R**	**125**	**8.24**	**36**	**−85**	**11**
	Occipital_Sup_R		6.88	24	–91	20
	Cuneus_R		6.76	15	–97	14
	**Lingual_L**	**72**	**8.16**	**0**	**−79**	**−1**
	**Frontal_Inf_Tri_L**	**612**	**8.08**	**−48**	**20**	**17**
	**Precentral_L**	**77**	**7.82**	**−48**	**−4**	**50**
	**Temporal_Mid_L**	**131**	**7.70**	**−48**	**−40**	**2**
	**Frontal_Sup_2_L**	**235**	**7.00**	**−12**	**29**	**53**
	Frontal_Sup_2_L		6.32	–9	53	35
	Supp_Motor_Area_L		6.12	–6	17	59
	**ParaHippocampal_L**	**201**	**6.76**	**−27**	**−31**	**−19**
	Hippocampus_L		6.70	–21	–16	–16
	**SupraMarginal_L**	**30**	**6.38**	**−54**	**−43**	**23**
	**Temporal_Inf_R**	**56**	**6.14**	**45**	**−61**	**−7**
	**Precentral_R**	**16**	**5.87**	**54**	**−1**	**44**
	**Occipital_Inf_L**	**6**	**4.85**	**−42**	**−67**	**−4**
MO and SP	**Occipital_Mid_R**	**109**	**8.11**	**39**	**−82**	**14**
	Occipital_Sup_R		6.78	24	–91	20
	Cuneus_R		5.86	15	–97	14
	**Frontal_Inf_Tri_L**	**609**	**8.08**	**−48**	**20**	**17**
	**Lingual_L**	**59**	**7.94**	**0**	**−79**	**−4**
	**Precentral_L**	**77**	**7.82**	**−48**	**−4**	**50**
	**Frontal_Sup_Medial_L**	**119**	**6.32**	**−9**	**29**	**53**
	Supp_Motor_Area_L		6.12	–6	17	59
	Supp_Motor_Area_R		6.03	3	5	65
	**Fusiform_L**	**38**	**6.22**	**−27**	**−34**	**−22**
	**Frontal_Sup_2_L**	**23**	**6.13**	**−9**	**53**	**35**
	**Precentral_R**	**19**	**5.87**	**54**	**−1**	**44**
	**Temporal_Inf_R**	**40**	**5.74**	**45**	**−61**	**−7**
	**Hippocampus_L**	**10**	**5.66**	**−18**	**−16**	**−16**
	**Insula_R**	**2**	**4.96**	**51**	**8**	**−7**
	**Temporal_Mid_L**	**1**	**4.78**	**−48**	**−40**	**−1**
	**Occipital_Mid_L**	**1**	**4.68**	**−39**	**−67**	**−1**
DO and SP	**Lingual_R**	**74**	**8.57**	**3**	**−79**	**−4**
	**Occipital_Mid_R**	**125**	**8.24**	**36**	**−85**	**11**
	Occipital_Sup_R		6.88	24	–91	20
	Cuneus_R		6.76	15	−97	14
	**Precentral_L**	**68**	**7.82**	**−48**	**−4**	**50**
	**Frontal_Inf_Tri_L**	**434**	**7.27**	**−42**	**26**	**5**
	Frontal_Inf_Orb_2_L		6.77	–45	23	−7
	**Temporal_Inf_R**	**57**	**6.14**	**45**	**−61**	**−7**
	**Supp_Motor_Area_L**	**90**	**6.12**	**−6**	**17**	**59**
	Supp_Motor_Area_R		6.03	3	5	65
	**Precentral_R**	**19**	**5.87**	**54**	**−1**	**44**
	**Insula_R**	**5**	**5.54**	**48**	**5**	**−7**
	**Occipital_Inf_L**	**5**	**4.85**	**−42**	**−67**	**−4**
	**Frontal_Sup_2_L**	**1**	**4.68**	**−15**	**35**	**50**
VMW and SP	**Occipital_Mid_R**	**74**	**8.24**	**36**	**−85**	**11**
	**Frontal_Inf_Tri_L**	**325**	**7.33**	**−45**	**26**	**5**
	Frontal_Inf_Orb_L		6.98	−42	26	−7
	**Frontal_Sup_L**	**183**	**7**	**−12**	**29**	**53**
**Conjunction of all four inner speech conditions (MS, MO, DO, VMW)**						
	Supp_Motor_Area_L		6.12	–6	17	59
	Supp_Motor_Area_R		6.03	3	5	65
	**Temporal_Inf_R**	**57**	**6.14**	**45**	**−61**	**−7**
	**Precentral_L**	**5**	**4.97**	**−45**	**8**	**47**
	**Precentral_R**	**3**	**4.94**	**51**	**2**	**47**
	**Occipital_Inf_L**	**6**	**4.85**	**−42**	**−67**	**−4**
MS and MO and DO and VMW	**Supp_Motor_Area_R**	**556**	**9.86**	**6**	**11**	**65**
	Supp_Motor_Area_L		8.78	–6	17	47
	Cingulum_Mid_L		8.24	–9	17	38
	**Insula_R**	**144**	**8.90**	**42**	**11**	**2**
	**Frontal_Inf_Orb_L**	**651**	**8.53**	**−45**	**17**	**−7**
	Frontal_Inf_Oper_L		8.41	–51	11	5
	Frontal_Inf_Tri_L		6.70	–48	38	2
	**Occipital_Mid_R**	**153**	**8.11**	**39**	**−82**	**14**
	Temporal_Mid_R		6.52	48	−73	8
	Temporal_Inf_R		5.74	45	−61	−7
	**Precentral_R**	**10**	**5.85**	**51**	**5**	**44**
	**Caudate_R**	**1**	**4.98**	**18**	**8**	**11**
	**Occipital_Mid_L**	**6**	**4.95**	**−39**	**−70**	**−1**
	**Caudate_L**	**3**	**4.84**	**−15**	**8**	**8**
	**Caudate_R**	**1**	**4.81**	**15**	**14**	**2**
**Conjunction of all five conditions (MS, MO, DO, VMW, SP)**						
ISS and ISO and IMA and VMW and SP	**Occipital_Mid_R**	**72**	**8.11**	**39**	**−82**	**14**
	**Frontal_Inf_Tri_L**	**310**	**7.27**	**−42**	**26**	**5**
	Frontal_Inf_Orb_L		6.77	–45	23	–7
	Frontal_Inf_Tri_L		6.52	−51	17	8
	**Supp_Motor_Area_L**	**89**	**6.12**	**−6**	**17**	**59**
	Supp_Motor_Area_R		6.03	3	5	65
	**Temporal_Inf_R**	**40**	**5.74**	**45**	**−61**	**−7**
	**Precentral_R**	**3**	**4.94**	**51**	**2**	**47**
	**Occipital_Mid_L**	**1**	**4.68**	**−39**	**−67**	**−1**
**Conjunction of SP and all inner speech conditions (MS, MO, DO, VMW) grouped together**						
4IS and SP	**Lingual_R**	**81**	**8.57**	**3**	**−79**	**−4**
	**Occipital_Mid_R**	**124**	**8.24**	**36**	**−85**	**11**
	Occipital_Sup_R		6.88	24	–91	20
	Cuneus_R		6.76	15	−97	14
	**Frontal_Inf_Tri_L**	**623**	**8.08**	**−48**	**20**	**17**
	Frontal_Inf_Tri_L		7.98	–51	35	14
	Frontal_Inf_Tri_L		7.64	–45	29	8
	**Precentral_L**	**77**	**7.82**	**−48**	**−4**	**50**
	**Frontal_Sup_L**	**235**	**7.00**	**−12**	**29**	**53**
	Frontal_Sup_L		6.32	–9	53	35
	Supp_Motor_Area_L		6.12	−6	17	59
	**Temporal_Mid_L**	**82**	**6.90**	**−48**	**−40**	**−1**
	**SupraMarginal_L**	**25**	**6.79**	**−51**	**−43**	**23**
	**Hippocampus_L**	**86**	**6.70**	**−21**	**−16**	**−16**
	ParaHippocampal_L		6.32	–30	–31	–19
	**Temporal_Inf_R**	**57**	**6.14**	**45**	**−61**	**−7**
	**Precentral_R**	**19**	**5.87**	**54**	**−1**	**44**
	**Insula_R**	**6**	**5.54**	**48**	**5**	**−7**
	**Temporal_Inf_L**	**11**	**5.22**	**−48**	**−49**	**−16**
	**Occipital_Inf_L**	**6**	**4.85**	**−42**	**−67**	**−4**

*Multiple peaks in each cluster are presented at *p* < 0.05 FWE correction. Main clusters are represented in bold font, with their extent size provided. Sub-clusters are represented in regular font.*

**FIGURE 7:**
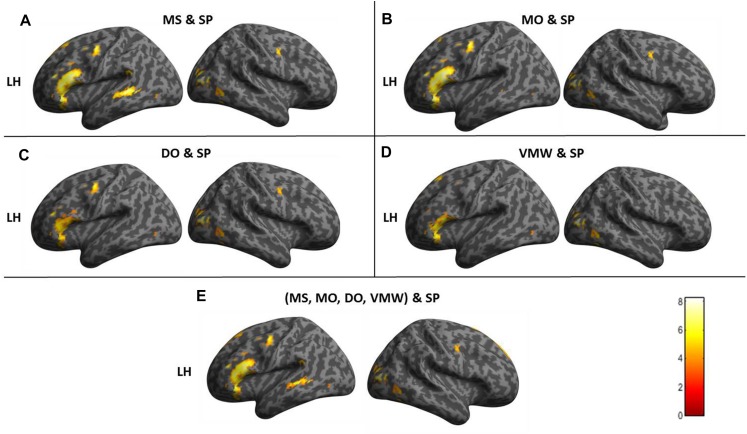
Conjunctions between each inner speech condition and SP projected on the surface of an inflated standard structural scan. **(A)** MS and SP. **(B)** MO and SP. **(C)** DO and SP. **(D)** VMW and SP. **(E)** (MS, MO, DO, VMW) and SP. All *p* < 0.05, FWE correction.

The conjunction between MS and SP (Figure 7A) confirmed that the left IFG, SFG, MTG/STG, SMA, SMG, hippocampus, bilateral PM cortex, and occipital/posterior MTG were recruited by both conditions. The conjunction between MO and SP (Figure 7B) yielded activation in left IFG, SFG, MTG, and hippocampus, as well as bilateral SMA, PM, and occipital/posterior MTG, thus revealing a weaker middle temporal cortex activation.

The conjunction between DO and SP (Figure 7C) yielded activation in left IFG, SFG, bilateral PM, SMA, right insula and bilateral occipital/posterior MTG but no middle temporal cortex activation.

The conjunction between VMW and SP (Figure 7D) yielded activation in left IFG, SFG, bilateral PM, SMA, and occipital/posterior MTG but no middle temporal cortex activation.

Conjunctions between all four inner speech conditions (MS, MO, DO, VMW), between all five conditions (MS, MO, DO, VMW, SP), and between all inner speech conditions grouped together and SP are listed in Table 2. Commonly activated regions in all four inner speech conditions (MS, MO, DO, VMW) and in all five conditions (MS, MO, DO, VMW, SP) include the left IFG, and bilateral SMA, but do not include the auditory cortex. The regions that show a conjunction of activity in SP and all inner speech conditions grouped together are illustrated in Figure 7E. In addition to left IFG and SMA, they include left supramarginal and middle temporal gyri.

**FIGURE 8:**
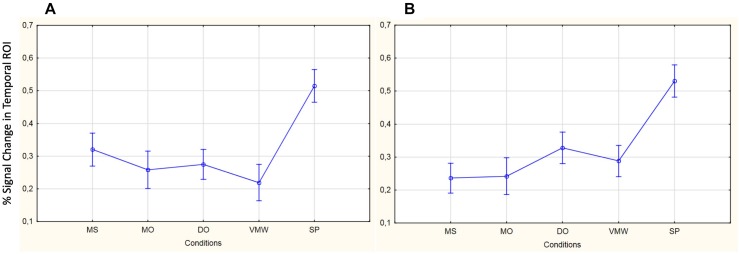
Percentage of signal change (%SC) in the temporal ROI including superior and middle temporal gyri, across the five experimental conditions (MS, MO, DO, VMW, and SP). **(A)** Left temporal ROI. **(B)** Right temporal ROI.

To further examine the degree of auditory activation in the different conditions, we extracted the %SC within a large temporal ROI including left Superior and Middle Temporal gyri (anatomically defined using AAL), in each hemisphere. The values are displayed in Figure 8 for each of the 5 conditions, in the left and right hemispheres. For each hemisphere, a one-way ANOVA was run on the %SC with condition as a factor. In the left ROI, results showed that the %SC in the SP condition was significantly different from each of the inner speech conditions (*p* < 0.001), with higher left temporal activation in SP than in each of the inner speech conditions. In addition, the MS condition was significantly different from VMW (*F*(1,23) = 7.92, *p* < 0.001), with higher left temporal activation in MS than VMW. In the right ROI, the %SC in the SP condition was significantly higher than in each of the inner speech conditions (*p* < 0.001). In addition, the %SC in the right ROI in the DO condition was significantly higher than in MS (*F*(1,23) = 16.11, *p* < 0.001) and MO (*F*(1,23) = 16.72, *p* < 0.001) and the %SC in the right ROI was higher in VMW than MS (*F*(1,23) = 5.96, *p* = 0.02).

#### Contrasts Between Conditions: Dialogality and Intentionality Dimensions

Contrasts between MS and MO, MO and DO, and VMW and MS are presented in Table 3, all for *p* < 0.05, FWE correction, except for the contrasts between MS and MO (*p* < 0.001, uncorrected).

**TABLE 3:** Contrasts between inner speech conditions.

**Contrast**				**MNI** **coordinates**		
				
	**Region label**	**Extent**	***t*-value**	***x***	***y***	***z***
**Contrast between MS and MO**						
MS > MO	**Cingulate_Mid_L**	**221**	**5.083**	**−18**	**−31**	**38**
	Postcentral_L		4.660	–27	–43	47
	Parietal_Sup_L		4.079	–33	–55	62
	**Frontal_Sup_2_L**	**140**	**4.867**	**−12**	**41**	**38**
	Frontal_Sup_Medial_L		3.870	–3	29	35
	Frontal_Sup_Medial_L		3.860	–6	41	23
	**Occipital_Mid_R**	**12**	**4.450**	**30**	**−88**	**17**
	**Frontal_Sup_Medial_L**	**17**	**3.975**	**−3**	**62**	**32**
	**Frontal_Inf_Tri_L**	**9**	**3.840**	**−57**	**20**	**20**
	**Lingual_L**	**10**	**3.585**	**−18**	**−58**	**−4**
	**Frontal_Sup_Medial_L**	**4**	**3.552**	**−6**	**47**	**50**
	**Occipital_Mid_L**	**4**	**3.495**	**−21**	**−94**	**2**
	**Caudate_L**	**7**	**3.454**	**−12**	**17**	**11**
	**Frontal_Inf_Tri_L**	**1**	**3.451**	**−54**	**26**	**26**
	**Fusiform_L**	**13**	**3.450**	**−33**	**−52**	**−16**
	**Frontal_Mid_2_L**	**1**	**3.423**	**−42**	**44**	**26**
	**Cingulate_Mid_L**	**5**	**3.409**	**0**	**−7**	**41**
	**Occipital_Sup_R**	**3**	**3.342**	**27**	**−76**	**38**
	**ParaHippocampal_L**	**10**	**3.312**	**−18**	**−37**	**−13**
	**Cerebellum_6_R**	**1**	**3.285**	**9**	**−79**	**−19**
	**Frontal_Inf_Orb_2_L**	**1**	**3.271**	**−39**	**23**	**−10**
	**Temporal_Inf_L**	**3**	**3.261**	**−45**	**−46**	**−10**
	**Frontal_Sup_Medial_L**	**3**	**3.260**	**−9**	**26**	**56**
	**Cerebellum_Crus1_R**	**1**	**3.210**	**45**	**−58**	**−28**
	**Frontal_Inf_Orb_2_L**	**2**	**3.201**	**−42**	**29**	**−10**
	**Parietal_Sup_R**	**1**	**3.198**	**18**	**−55**	**71**
MO > MS	**Putamen_R**	**163**	**−4.510**	**21**	**2**	**8**
	**Frontal_Mid_2_R**	**122**	**−4.130**	**36**	**41**	**11**
	Frontal_Inf_Oper_R		–3.960	60	14	11
	Frontal_Inf_Tri_R		–3.370	48	29	8
	**Frontal_Inf_Tri_R**	**19**	**−4.030**	**33**	**17**	**23**
	**Thalamus_R**	**62**	**−4.000**	**15**	**−19**	**−1**
	Pallidum_R		−3.790	27	−13	−4
	**Supp_Motor_Area_R**	**15**	**−3.600**	**9**	**−4**	**53**
	**SupraMarginal_R**	**12**	**−3.430**	**63**	**−25**	**29**
**Contrast between MO and DO**						
MO > DO	**Supp_Motor_Area_L**	**134**	**6.564**	**−9**	**17**	**68**
	Supp_Motor_Area_L		6.280	–6	20	50
	Cingulate_Mid_L		6.170	–6	26	35
	**Frontal_Inf_Tri_L**	**78**	**5.878**	**−33**	**26**	**−1**
	**Putamen_L**	**42**	**5.614**	**−21**	**5**	**11**
	**Frontal_Inf_Oper_L**	**16**	**5.437**	**−51**	**11**	**5**
	**Frontal_Inf_Tri_L**	**29**	**5.424**	**−54**	**17**	**20**
	**Thalamus_L**	**7**	**4.906**	**−6**	**−13**	**5**
	**Pallidum_R**	**4**	**4.886**	**9**	**2**	**−4**
	**Cingulate_Mid_R**	**1**	**4.685**	**12**	**23**	**29**
DO > MO	**Parietal_Inf_R**	**2083**	**−7.51**	**39**	**−46**	**41**
	Cingulate_Mid_R		–7.47	15	–40	35
	Precuneus_R		–7.35	12	–58	41
	**Frontal_Sup_2_R**	**187**	**−7.40**	**24**	**23**	**44**
	**Frontal_Mid_2_R**	**41**	**−5.63**	**45**	**23**	**41**
	**Frontal_Mid_2_R**	**39**	**−5.63**	**42**	**41**	**5**
	Frontal_Mid_2_R		–4.82	30	47	2
	**Frontal_Sup_2_R**	**5**	**−5.150**	**24**	**56**	**11**
	**Temporal_Mid_R**	**19**	**−5.140**	**57**	**−52**	**−1**
	**Frontal_Inf_Tri_R**	**6**	**−5.090**	**33**	**14**	**23**
	**Angular_L**	**7**	**−4.860**	**−42**	**−58**	**41**
	**Temporal_Mid_R**	**1**	**−4.690**	**51**	**−43**	**−7**
**Contrast between VMW and MS**						
MS > VMW	**Fusiform_L**	**2593**	**10.155**	**−33**	**−43**	**−22**
	Calcarine_R		9.765	18	–61	5
	**Precentral_L**	**299**	**8.858**	**−48**	**−4**	**50**
	Precentral_L		5.110	–33	–19	50
	**Supp_Motor_Area_L**	**103**	**6.670**	**−3**	**2**	**62**
	**Frontal_Inf_Tri_L**	**172**	**6.271**	**−51**	**35**	**17**
	Frontal_Inf_Oper_L		6.229	–45	14	20
	**Putamen_L**	**34**	**5.989**	**−24**	**5**	**11**
	**Frontal_Inf_Orb_2_L**	**28**	**5.810**	**−39**	**29**	**−13**
	**Temporal_Mid_L**	**47**	**5.793**	**−48**	**−40**	**2**
	**Insula_L**	**59**	**5.713**	**−36**	**−25**	**20**
	**Caudate_L**	**6**	**5.459**	**−15**	**−28**	**23**
	**Cingulate_Mid_L**	**6**	**5.160**	**−6**	**14**	**38**
	**Precentral_R**	**2**	**5.096**	**57**	**−1**	**41**
	**Precentral_R**	**4**	**5.001**	**30**	**−19**	**71**
	**Insula_L**	**1**	**4.821**	**−36**	**−4**	**14**
VMW > MS	**Parietal_Inf_R**	**2701**	**−11.720**	**36**	**−43**	**41**
	Precuneus_R		–9.540	12	–67	50
	**Frontal_Sup_2_R**	**1749**	**−9.000**	**24**	**56**	**14**
	Frontal_Mid_2_R		–8.600	39	47	14
	**Frontal_Mid_2_L**	**103**	**−7.100**	**−30**	**56**	**5**
	**Frontal_Sup_2_L**	**29**	**−5.890**	**−27**	**32**	**38**
	**Insula_R**	**32**	**−5.780**	**33**	**14**	**−10**
	**Cingulate_Ant_R**	**16**	**−5.440**	**9**	**38**	**17**
	**Cerebelum_Crus1_L**	**6**	**−5.280**	**−27**	**−67**	**−31**
	**Frontal_Sup_Medial_R**	**10**	**−5.270**	**6**	**59**	**2**
	**Angular_L**	**9**	**−5.270**	**−51**	**−58**	**38**
	**Thalamus_R**	**3**	**−5.060**	**15**	**−25**	**11**
	**Frontal_Sup_2_L**	**9**	**−4.970**	**−24**	**2**	**53**
	**Frontal_Sup_Medial_R**	**3**	**−4.890**	**6**	**47**	**−1**
	**Insula_R**	**1**	**−4.790**	**39**	**−7**	**−10**
	**Temporal_Mid_R**	**2**	**−4.780**	**54**	**−61**	**2**

*Multiple peaks in each cluster are presented at *p* < 0.05 FWE correction (except for MS vs. MO, *p* < 0.001 uncorrected). Main clusters are represented in bold font, with their extent size provided. Sub-clusters are represented in regular font.*

##### Dialogality dimension: voice control in inner speech

**FIGURE 9:**
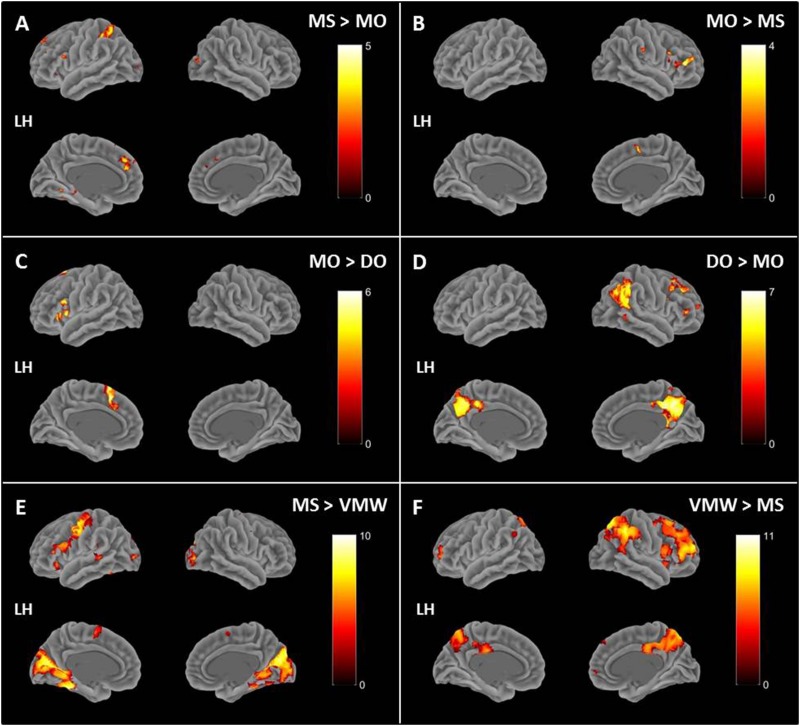
Contrasts between conditions rendered on a standard 3D brain provided by BSPMview. **(A)** MS > MO. **(B)** MO > MS. **(C)** MO > DO. **(D)** DO > MO. **(E)** MS > VMW. **(F)** VMW > MS. All *p* < 0.05, FWE correction, except for MS > MO and MO > MS (*p* < 0.001, uncorrected).

The contrasts between MS and MO suggest that covertly using someone else’s voice (MO) vs. one’s own voice (MS) resulted in an increased involvement of the right hemisphere (Figures 9A,B). More specifically, in the MS > MO contrast, greater left hemisphere recruitment was observed, with activation in left IFG (BA 45), SFG (BA 8), medial SFG (BA 8, 32), middle cingulate, postcentral, and superior parietal lobule (BA 7). In MO > MS, greater right hemisphere involvement was found, with activation in right IFG (BA 44, 45), SMA, MFG (BA 10) and inferior parietal lobule (BA 40).

##### Dialogality dimension: perspective control in inner speech

Perspective switching, from monologal other-voice to dialogal other-voice was examined through the MO vs. DO contrasts (Figures 9C,D). In MO > DO, greater activation was observed in left IFG (BA 44), SMA, and ACC and in DO > MO, we found a greater recruitment of right IFG (BA 44), MFG (BA 8, 10, 46), SFG (BA 8), as well as bilateral inferior (BA 39, 40) parietal lobules, precuneus and posterior cingulate cortex. This last contrast indicates an increase in right hemisphere activation in DO relative to MO.

**FIGURE 10:**
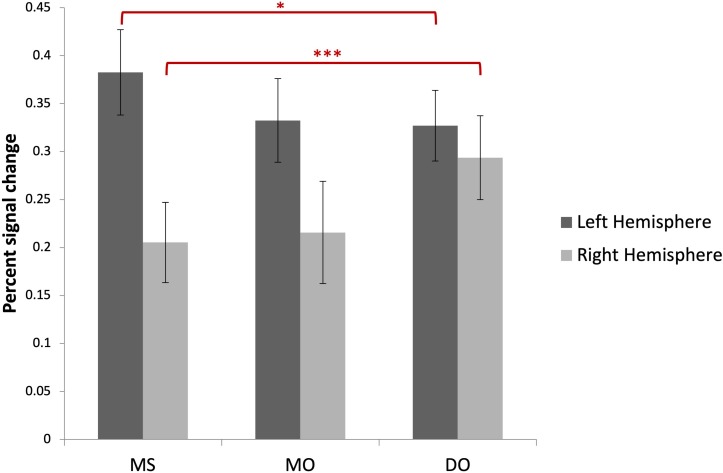
Percent signal changes computed on an anatomically defined set of ROIs (inferior frontal and precentral gyri, SMA, superior and middle temporal gyri as well as inferior and superior parietal cortex), in left and right hemispheres, for the MS, MO, and DO conditions. In these ROIs, from MS to DO, a significant decrease (^∗^) is observed in left hemisphere activation associated with a significant increase (^∗∗∗^) in right hemisphere recruitment.

To quantify the increase in right hemisphere involvement and relative disengagement of left hemisphere, the %SC values within a symmetrical left-right set of ROIs were submitted to an ANOVA crossing the factors hemispheric lateralization (right, left) and condition (MS, MO, DO). As illustrated in Figure 10, results showed a main effect of lateralization (*F*(1,23) = 55.63, *p* < 0.001) and a significant lateralization-by-condition interaction (*F*(2,46) = 18.63, *p* < 0.001), indicating that condition affected hemispheric lateralization. Further tests showed that %SC values in MS and DO were significantly different, both for the right (*F*(1,23) = 17, *p* < 0.001) and the left (*F*(1,23) = 5.08, *p* = 0.03) hemispheres, with more left lateralization for MS than DO and more right lateralization for DO than MS. The difference between MS and MO was not statistically significant neither for the right (*F*(1,23) = 0.12, *p* = 0.73), nor for the left (*F*(1,23) = 3.73, *p* = 0.06) hemispheres.

##### Intentionality dimension

Switch from intentional to unintentional inner speech was examined through the MS vs. VMW contrasts (Figures 9E,F), since the VMW condition, according to participants, contained verbal episodes. In MS > VMW, greater activation was observed in left SMA, primary motor, IFG (BA 44, 45, 47), insula, MTG/STG (BA 21, 22), SMG, ACC, putamen, caudate, and bilateral PM. In VMW > MS, greater activation was observed in right inferior parietal (BA 7, 40), precuneus, IFG (BA 47), SFG (BA 9, 10), MFG (BA 10), insula, ACC, thalamus, left SFG (BA 6). Some of these activations might reflect the involvement of the Default Mode Network (DMN, Buckner et al., 2008). In order to further describe the specificity of the VMW condition relative to the DMN, the participants were split into two groups (High-verbal and Low-verbal) based on their amount of reported verbal episodes during the VMW condition (below and above the median). A two-sample *t*-test was used to compare the two groups on this condition. Compared to Low-verbal, High-verbal participants did not show any additional activation. However, the opposite contrast showed that the Low-verbal participants showed more activation of the dorsomedial prefrontal cortex than the High-verbal participants (*p* < 0.05, FWE corrected), as detailed in Table 4.

**TABLE 4:** Contrasts between the two groups of participants (Low verbal > High verbal) in the VMW condition (*p* < 0.05 FWE correction).

**Contrast name**				**MNI** **coordinates**		
				
	**Region label**	**Extent**	***t*-value**	***x***	***y***	***z***
Low verbal > High verbal	Frontal_Sup_Medial_L	16	7.14	0	47	38

## Discussion

Our fMRI protocol allowed us to investigate varieties of inner speech along dialogality and intentionality dimensions, in the aim of examining the validity of the neuroanatomical correlates posited in the ConDialInt model. To explore dialogality, three controlled inner speech conditions were elicited. This allowed us to compare monologal inner speech with own and other voices, probing for prosodic and voice aspects of dialog. The comparison between monologal and dialogal inner speech (both produced with other voice), allowed us to reveal aspects specifically associated with perspective shifting. To explore intentionality, willful inner speech was compared with mind wandering, during which verbal activity was reported.

### Intentional Monologal Expanded Inner Speech: The Inner Voice as an Efference Copy Prediction

Occipital activation in all conditions can be related to the visual processing required at the beginning of each trial when the pictures are presented. The pattern of activation observed in the SP condition (compared with the baseline or in conjunction with inner speech conditions) was consistent with previous studies on auditory sentence perception and argues in favor of speech perception theories that include a premotor component (see e.g., Friederici, 2011 for a review).

The contrast between MS and baseline (as well as the conjunction between MS and SP) indicates that intentional monologal own voice inner speech was associated with left hemisphere activation in regions compatible with the predictive control scheme assumed in the ConDialInt model. The contrast between MS and baseline reveals prefrontal cortex activation, in MFG and SFG, regions which have been associated with cognitive control (Ridderinkhof et al., 2004). It has been suggested that the orbitofrontal cortex plays an inhibitory role during motor imagery (Jeannerod, 2001). The recruitment of the orbitofrontal cortex could therefore indicate that inhibitory processes are engaged, to prevent overt production. More detailed effective connectivity or sEEG data are needed, however, to assess whether this orbitofrontal cortex activation does reflect inhibitory influence on areas involved at the various stages of language production. An alternative account, which does not appeal to inhibitory processes, could be that the highest processing levels are too weakly activated for the last stage (motor execution) to be launched. The contrast between MS and baseline also shows activation in the hippocampus and posterior MTG, which were presumably related to conceptualization. The recruitment of IFG can be associated to formulation and articulatory planning, whereas SMG activation can be related to phonetic goal integration. The activation of the right cerebellum is consistent with the recruitment of controller/predictor models. We can speculate that the phonetic goal issuing from the SMG was sent to a controller in the right cerebellum, which converted it into a motor specification. This motor specification was then coordinated with ongoing motor actions via the recruitment of left IFG, bilateral SMA and PM cortex, resulting in motor commands. An efference copy of these commands could then have been sent to a predictor model in cerebellum. We have argued above for the role of the cerebellum in both motor command preparation (controller) and sensory experience prediction (predictor), with perhaps a distinction between anterior and posterior lobes. Our data do not allow us to assess whether this distinctive pattern of activation occurred, however, given that the field of MR acquisition provided full coverage of the cerebrum but did not cover the entire cerebellum. The observed cluster of activation crossing posterior STG and MTG suggests that auditory percepts were experienced. The recruitment of the right cerebellum together with the auditory activation is compatible with the hypothesis made in the ConDialInt model that the cerebellar predictor model issues predicted sensory signals processed by the auditory cortex. More refined connectivity analyses or neuroimaging data with better temporal resolution could further test this hypothesis. The ConDialInt model posits an attenuation mechanism for self-generated auditory experience relative to externally generated sounds. Our data are consistent with this hypothesis, since less STG/MTG activation was observed during MS than SP. In their study of elicited vs. spontaneous inner speaking, Hurlburt et al. (2016) even found a deactivation of Heschl’s gyrus during elicited inner speech compared with the baseline (not only compared with speech perception). They used a region of interest (ROI) analysis centered on Heschl’s gyrus, however, and do not report whole-brain analysis results. Agnew et al. (2013) have observed an anterior-posterior division of activity profiles within the STG, where anterior fields are suppressed during (aloud or silent) motor output, whereas posterior fields remain engaged. It is possible that there was some STG/MTG activation during intentional inner speech in Hurlburt et al.’s study, but the restricted ROI analysis may have missed it. Therefore, the neural network that was observed in the present study supports the claim that intentional monologal inner speech involves the inhibited production of motor commands, generated in left frontal regions. Efference copies of the commands would be processed by the cerebellar predictor, giving rise to a sensory experience, the inner voice, albeit a weaker one than during actual speech perception. The ConDialInt model conjectures that the predictor should issue both auditory and somatosensory responses, later integrated into a supramodal representation, via the temporo-parietal junction (TPJ). Except in the MS vs. MO (uncorrected) contrast, we could not observe any somatosensory activation during any of the intentional tasks. This could be due to a lack of power, but we cannot conclude that multisensory representations are indeed at play. The fact that we did register SMG activation (with a cluster encompassing the TPJ) is compatible with an integration process after auditory response, however.

Intentional monologal inner speech with someone else’s voice (MO) or intentional dialogal inner speech with someone else’s voice (DO) also resulted in networks of IFG and motor activations consistent with our predictive account. The lack of superior temporal gyrus activation can be attributed to the fact that, during MO and DO, internal models are less accurate than during MS, and presumably generate more precarious auditory predictions. This could explain the lesser auditory cortex activation. This account is supported by the participants’ subjective experience of a fainter voice percept in these more cognitively demanding conditions (see also Shergill et al., 2001).

### Dialogality Dimension: Neural Correlates of Producing Another Voice

Along the dialogality dimension, covertly using someone else’s voice (MO) vs. one’s own voice (MS), in a monolog, resulted in a marginally significant decrease of left hemisphere activation in the ROIs. More specifically, greater left IFG, postcentral and superior parietal activation was observed in MS > MO, whereas greater right IFG and parietal activation was detected in MO > MS (uncorrected contrasts). In addition, the cerebellar activation observed in MS was reduced in MO. The MO condition required a mental shift in fundamental frequency range, and perhaps even in voice quality, as the avatar’s voice to be imitated was extremely high-pitched. Some prosodic fluctuations, and especially those related to affective, emotional or attitudinal aspects are considered to involve the right hemisphere, typically the right inferior frontal gyrus (Baum and Pell, 1999; Lœvenbruck et al., 2005; Pichon and Kell, 2013). Thus, in the framework of predictive control, the present results suggest that mentally imitating a high-pitched voice requires to modify the controller/predictor pair, at least at the articulatory planning stage. The self-adapted controller/predictor models that are suspected to involve the right cerebellum in MS are not adequate, and right frontal region recruitment seems to take place instead. Participants reported that the MO task was more difficult than MS. An alternative interpretation could be that increased cognitive load resulted in the recruitment of contralateral homologous regions. The fact that MS resulted in greater left postcentral and superior parietal activation than MO could suggest that the somatosensory representations evoked when inner speaking with self-voice are stronger that when a different voice is used.

When comparing DO relative to MS, our analyses on the set of frontal, temporal and parietal ROIs (Figure 10), revealed a significant increase in the recruitment of the right hemisphere (also observed on the temporal ROI alone, Figure 8) together with a significant decrease in left hemisphere activation. Crucially, the DO > MS contrast showed activity in right IFG, MTG and SMG. Similar right hemisphere activation was found in Shergill et al.’s (2001) fMRI study, in six participants who were examined during (first, second and third person) auditory verbal imagery. Linden et al. (2011) also found significant right hemisphere activation in fronto-temporal regions during voluntary auditory imagery. These findings also chime with the fMRI data obtained by Sommer et al. (2008). They compared the cerebral activation of patients diagnosed with schizophrenia while they experienced auditory verbal hallucination (AVH) and while they produced normal inner speech. They found that the main difference between the two conditions was lateralization, with a predominant engagement of the right inferior frontal region during AVH. An influential account formulates AVH as inner speech misattributed to an external source due to a dysfunction in efference copy and predictive control mechanisms (Feinberg, 1978; Frith, 1992; Jones and Fernyhough, 2007b; but see Gallagher, 2004). Rapin et al. (2013, 2016) have argued that this account leaves several questions open, however. First, with this rationale, all inner speech should be mistaken as coming from an external agent, yet patient interviews show that this is not the case (Larøi and Woodward, 2007; Aleman and Larøi, 2008). Secondly, this model does not describe how “other” voices are heard, yet patients with schizophrenia often report that they can precisely identify the voice they hear as being clearly that of someone they know and as addressing them in the second person (Hoffman et al., 2011). In our view, AVH does not result from a disruption in MS but from MO or rather DO. In the Sommer et al. (2008) study, when patients experienced AVH, right IFG activation occurred, just like when the participants of the present study imagined the avatar addressing them. The lack of agency felt by the patients could be due to a fawlty agency attribution mechanism when other-adapted controller/predictor models are used. If controller and predictor, for instance, are not symmetrical or temporally misaligned, then the prediction could differ from the desired signal. This would make the predicted auditory experience feel alien, leading to a misattribution to an external source. This interpretation is consistent with an fMRI study by Shergill et al. (2000) on eight patients with schizophrenia who had had experiences of AVH but were in remission at the time of study. They found that the activation pattern of patients during inner speech was not different from that of control healthy subjects, but that attenuated activation was evident in posterior cerebellar cortex, hippocampi, and lenticular nuclei bilaterally and the right thalamus, middle and superior temporal cortex, and left nucleus accumbens, during auditory verbal imagery (similar to what we refer to here as DO). This implies that in patients with a history of AVH, auditory verbal imagery (DO), but not monologal self-voice inner speech (MS), is associated with an atypical neural activation pattern. This pattern, when exacerbated in pathological condition, may contribute to the spurring of AVH.

### Dialogality Dimension: Neural Correlates of Imagining Another Voice Speaking (Third-Perspective Taking)

To study perspective switching by itself, the contrast between MS and DO is not adequate, because a change in voice (self-voice vs. other-voice) is confounded with a change in perspective (self speaking vs. other speaking). We therefore examined the contrast between MO and DO, since both conditions required the generation of another voice. Relative to MO, DO additionally recruited the right IFG, MFG, SFG, right superior and inferior parietal lobules as well as bilateral precuneus and posterior cingulate cortex. The recruitment of right frontal region seems therefore even more important in DO than in MO. As argued above, right frontal activation can be related to prosody control at the articulatory planning stage, and this could mean that suprasegmental control is even more demanding in DO. It could alternatively suggest that increased cognitive load in DO, relative to MO, resulted in the recruitment of contralateral regions homologous to the regions associated with articulatory planning. The recruitment of right parietal cortex is consistent with several studies on perspective switching and imagination of others’ actions. Ruby and Decety (2001) found that imagining someone perform an action (what they refer to as third person perspective) involves the inferior parietal lobule, the precuneus, the posterior cingulate, and the frontopolar cortex. Tian et al. (2016) have examined the neural correlates of articulation imagery and hearing imagery. Articulation imagery consisted in imagining producing a syllable (/ba/ or /ki/) and can be considered as close to our MS condition. Hearing imagery consisted in imagining hearing those same syllables, produced by a (previously introduced) female speaker. The authors did not report any right parietal activation during hearing imagery. But their task was aimed at eliciting memory retrieval of previously heard syllables, and participants were specifically asked to minimize production. Therefore, the discrepancy between their results and our own can be explained by the different nature of the tasks. In their fMRI study of auditory imagery, Linden et al. (2011) did not find any parietal activation either. The participants’ task consisted in simply imagining one or several familiar voices speaking to them for a few seconds. Using a region of interest analysis, they observed bilateral activation in the superior temporal sulcus (the voice selective region). In addition, they found bilateral activation in IFG, SMA, ACC and cuneus. The lack of parietal activation could also be explained by the nature of the task, which resembles the hearing imagery task by Tian et al. (2016). Linden et al. (2011) state that the most common strategy for participants was to imagine voices of familiar people, such as family conversations or messages left on the phone. Therefore, participants may have been more strongly focusing on memory retrieval rather than actual verbal production with an allocentric perspective. Alderson-Day et al. (2016) used a novel fMRI paradigm in which matched scenarios elicited either monologal (speaking from a single perspective) or dialogal (dialogs between two people) inner speech. The contrast between dialogal and monologal inner speech revealed increased activation in STG bilaterally, left IFG and MFG, left precuneus, and right posterior cingulate. The observed precuneus and posterior cingulate activation converges with our results and those of studies on egocentric and allocentric perspective handling (see e.g., Ruby and Decety, 2001, or Blanke, 2012 for a review) and suggests that these regions are critically involved in perspective switching. Contrary to our own results, however, there was no increase in right IFG and MTG in dialogal inner speech compared with monologal inner speech in their study. The fact that their dialogal condition used several scenarios which involved different voices (a teacher, a job recruiter, a relative, the prime minister) whereas our MO and DO conditions involved one single high-pitched voice, could explain this discrepancy. The auditory experience related to a single caricatural voice may be easier to predict than the many sensations associated with many voices.

### Intentionality: Neural Correlates of Verbal Mind Wandering

Finally, along the intentionality dimension, when compared with the baseline, VMW displayed greater left hemisphere activation in SMA, together with bilateral IFG, insula, MFG, SMA, medial SFG, inferior and superior parietal cortex, precuneus, and left caudate, thalamus, and cerebellum. The activation of medial SFG, precuneus, posterior inferior parietal regions and lateral temporal cortex is compatible with the default mode network. The addition of the bilateral IFG and insula fits with the verbal quality of this mind wandering period. When the participants were split into Low-verbal vs. High-verbal groups, it was found that, compared with the High-verbal group, the Low-verbal group showed more activation in the dorsomedial prefrontal cortex, classically related to cognitive control (Venkatraman et al., 2009). This could suggest that for unintentional inner speech to occur, cognitive control should be turned down. Further data are required to confirm this result. The contrast between MS and VMW yielded an increase in right hemisphere involvement for VMW relative to MS. Increased activation was observed in left parieto-fronto-temporal regions in MS compared with VMW, whereas VMW yielded greater activation than MS in right parieto-fronto-temporal regions, as well as precuneus, ACC, and thalamus (see also the ROI analysis in temporal regions, Figure 8). Since an increase in right hemisphere activation was also observed in DO, this could suggest that the VMW condition may include periods of monologal as well as dialogal inner speech. This is consistent with the post-scan questionnaires: participants reported that they experienced verbal material, and this could be addressed to them or spoken by them. The occipital activation decreased in VMW with respect to MS. This is possibly due to the higher visual stimulation in the latter condition. In the MS condition, a new picture, with the associated word to define, was presented every 8 s, whereas in the VMW condition, a picture was presented only once, for 2 s, at the beginning of the trial and then the visually neutral rotating clock appeared. The left STG-MTG activation decreased in VMW compared with MS, just as it did for MO and DO, presumably reflecting the fainter auditory percepts in these conditions. Spontaneous inner speech, i.e., inner speaking episodes during a mind wandering session, was examined in Hurlburt et al.’s (2016) study cited above, using a ROI analysis focused on Heschl’s gyrus and the left IFG. Contrary to our results, compared with baseline, their spontaneous speech samples yielded increased activation in Heschl’s gyrus and no difference was observed in the left IFG. Although our participants were trained to report on spontaneous inner speech, they did not go through the thorough descriptive experience sampling and expositional interview process used in the Hurlburt et al. (2016) study. The five participants in Hurlburt et al.’s (2016) study had been extensively trained and received guidance to distinguish between spontaneous inner speaking (unintentional monologal inner speech) and spontaneous inner hearing (unintentional dialogal inner speech). Their data only concerns inner speaking, which was the most frequent of the spontaneous speech forms. The more limited training underwent by the participants in our own study probably reduces the validity of the reports. Yet, the observed left IFG activation during VMW suggests that participants did produce inner speech, at least in a semi-expanded form (LIFG is supposed to be already recruited at the formulation stage). It is somewhat surprising that the left IFG was not recruited in Hurlburt et al.’s (2016) spontaneous inner speaking samples. One explanation for the presence of left IFG in our data and the absence in theirs could lie in the different types of contrasts used. Whereas we compared the entire VMW condition with an implicit baseline, Hurlburt et al. (2016) contrasted spontaneous inner-speaking-dominant with spontaneous not-inner-speaking-dominant samples. DES samples rarely contain only one kind of experience, inner speaking may be accompanied with inner seeing or other phenomena (Hurlburt et al., 2013). Inner speaking occurrences were carefully selected using the DES method. Inner-speaking occurrences (20 of all 180 spontaneous samples, across the five participants) only included samples for which three interviewers unanimously rated that inner speaking was the predominant feature of the inner experience. These 20 samples were compared with 85 not-inner-speaking samples that were unanimously rated as not containing inner speaking. As acknowledged by the authors, it cannot be excluded that the absence of significant difference in left IFG activation during these two sets of samples could be due to a lack of power. The other difference between our findings and those of Hurlburt et al. (2016) lies in the pattern of temporal lobe activation. We have found a gradient of left temporal activation, from high STG-MTG involvement during SP to minimal activation during VMW via medium recruitment during MS, whereas Hurlburt et al. (2016) observe a strong activation in Heschl’s gyrus during spontaneous inner speech, and a deactivation during intentional inner speech. The fact that we observed such a weaker left auditory activation during VMW could be explained by the variety of inner speech at play. As mentioned, in Hurlburt et al.’s (2016) study, inner speaking occurrences were unanimously rated by three interviewers as containing inner speaking. Presumably, these instances were expanded forms of inner speech, with full inner production down to the articulatory planning stage and inner voice prediction. In our own study, participants reported any verbal material, which may have included full-fledged inner voice as well as less expanded forms. We did not select specific instances, but kept instead the entire VMW session. Some of the verbal forms experienced by our participants may therefore have been more condensed than the inner speaking samples selected in Hurlburt et al.’s (2016) study. Therefore, the reduced left auditory activation observed in the present study could be a result of higher condensation in the spontaneous speech observed (as the subjective reports presented in Figure 5 suggest). We did observe an increase in right temporal activation during VMW (and DO) relative to MS, however. This could suggest that VMW included dialogal inner speech occurrences, be they semi-condensed or expanded. Alternatively, our finding on the reduction of left temporal activation could be due to a lack of power and an insufficient number of spontaneous inner speech fragments, since verbal episodes were only transient during each VMW trial.

## Conclusion

On the basis of recent psycholinguistic and neuroimaging data combined with early introspective descriptions, we have proposed ConDialInt, a comprehensive neurocognitive model of inner speech, aiming to account for typical varieties.

We have presented an fMRI study in which we probed varieties of inner speech along dialogality and intentionality dimensions, in the aim of examining the neuroanatomical assumptions of the ConDialInt model. We designed several carefully controlled tasks specifically fit to compare inner speech along those two dimensions. The condensation dimension was also informally tackled.

Our findings support the predictive control hypothesis that expanded inner speech recruits speech production processes down to articulatory planning, resulting in a predicted signal, the inner voice, with auditory qualities. More specifically, the data are compatible with an account in which a supramodal phonetic goal, instantiated in the inferior parietal lobule, is presumably converted into motor commands that are inhibited by cognitive control signals originating from prefrontal cortex, so that no movement of the speech apparatus occurs. The specification of motor commands is supposed to involve a controller model that may be sustained by the right cerebellum, as well as further coordination processes handled by the left IFG, insula, and premotor cortex. An efference copy of the motor commands may be used by a predictor model supported by the right cerebellum, giving rise to auditory percepts handled in STG and MTG.

Along the dialogality dimension, covertly using an avatar’s voice with a high pitch, instead of one’s own voice, during monologal other-voice inner speech, recruited right hemisphere homologs of the regions involved in own-voice soliloquy. These right hemisphere regions are presumably associated with pitch control. The lesser cerebellar activation indicates that self-adapted controller/predictor models are inadequate in such a task. Changing perspective, from monologuing to imagining other speaking, was associated with activations in precuneus and parietal lobules, in addition to the pitch-control regions. In line with previous studies on imagination of others’ actions or others’ speech, we suggest that these regions play a crucial role in first-person and third-person perspective handling.

Finally, along the intentionality dimension, mind wandering with unintentional inner speech episodes was associated with bilateral inferior frontal activation and less activation in left temporal regions than intentional inner speech. This is coherent with the subjective evanescence quality reported by the participants and presumably reflects condensation processes. Whereas the intentional inner speech tasks all implied speech production down to articulatory planning and generation of an inner voice, the verbal episodes during the mind wandering trials were presumably less expanded. Yet the observation of left IFG activation in this condition does suggest that the initial stages of speech production were launched.

The ConDialInt model includes informed speculations on the neural correlates of the conceptualization, formulation and articulatory planning stages of inner speech. Although our data are consistent with these propositions, further studies are needed to test the model more thoroughly and to refine the descriptions. Several questions are still open. Most notably, we have made the hypothesis that the phonetic goal, generated from conceptualization and formulation, is in a supramodal format, that integrates somatosensory and auditory representations. We argue that this phonetic goal is formed within the IPL, before it is sent to the cerebellar controller and later to prefrontal and premotor regions. This is speculative and more refined neuroimaging or electrocorticography (EcoG) studies, with more precise temporal and spatial resolution, should help better describe the temporal sequence of cerebral activations between IPL, cerebellum and IFG-PM cortex. We have also assumed that both controller and predictor models are sustained by the cerebellum, based on recent findings on the double representation of the cerebral regions in the anterior and posterior lobes of the cerebellum. But the present fMRI data do not cover enough of the cerebellum to assess whether different parts of the cerebellum were involved. Furthermore, they do not allow us to test whether the assumed cortico-cerebello-cortical sequence of activation is appropriate. Our model conjectures that multisensory responses are the predicted outputs of internal predictors. Yet we mainly registered an auditory response and little somatosensory activity. Further studies are necessary to assess whether somatosensory activation can be detected. We also speculated that the auditory and somatosensory responses are integrated (via the TPJ) to form a supramodal response, comparable to the initial phonetic goal. This too needs to be better tested, by examining inferior parietal cortex activity in more detail. Furthermore, we have conjectured that the prefrontal activation observed is associated with inhibitory control (suppressing the motor output), as well as with executive control, related to monitoring one’s inner speech in intentional instances, and to holding different perspectives in dialogal varieties. Further studies should help disentangle between these different types of control. Moreover, we have speculated that the lack of left auditory cortex responses in the mind wandering condition was due to our participants producing more condensed varieties of inner speech during these trials. Unintentional inner speech is often reported as faint and evanescent, as if its auditory quality was dimmer or even absent. Given that another study did find a strong auditory response during spontaneous speech, further phenomenological and neuroimaging studies are needed to better describe the degree of expansion during unintentional inner speech. Whether or not expanded varieties of inner speech mostly arise during intentional inner speech remains an open question.

## Data Availability

The datasets generated for this study are available on request to the corresponding author.

## Ethics Statement

Each participant gave informed written consent and received 30€ for their participation. The study was approved by the local ethics committee (38RC14.304/ID-RCB: 2014-A01403-44).

## Author Contributions

All authors contributed to the conception and design of the study, discussion of the results, revision of the manuscript, and read and approved the submitted version. LR, RG, and CP collected the fMRI data. RG, CP, HL, MP-B, MB, and EC designed fMRI data analysis methods. RG, CH, CP, and EC performed the data analysis. HL wrote the first draft and revised version of the manuscript. RG wrote sections of the manuscript.

## Conflict of Interest Statement

The authors declare that the research was conducted in the absence of any commercial or financial relationships that could be construed as a potential conflict of interest.

## Footnotes

1 http://www.neurobs.com

2 https://www.fil.ion.ucl.ac.uk/spm/
